# Comparative structure activity and target exploration of 1,2-diphenylethynes in *Haemonchus contortus* and *Caenorhabditis elegans*

**DOI:** 10.1016/j.ijpddr.2024.100534

**Published:** 2024-03-19

**Authors:** Harrison T. Shanley, Aya C. Taki, Nghi Nguyen, Tao Wang, Joseph J. Byrne, Ching-Seng Ang, Michael G. Leeming, Nicholas Williamson, Bill C.H. Chang, Abdul Jabbar, Brad E. Sleebs, Robin B. Gasser

**Affiliations:** a Department of Veterinary Biosciences, Melbourne Veterinary School, Faculty of Science, The University of Melbourne, Parkville, Victoria, 3010, Australia; b Walter and Eliza Hall Institute of Medical Research, Parkville, Victoria, 3052, Australia; c Melbourne Mass Spectrometry and Proteomics Facility, The Bio21 Molecular Science and Biotechnology Institute, The University of Melbourne, Parkville, Victoria, 3010, Australia

**Keywords:** Anthelmintic discovery, *Haemonchus contortus*, *Caenorhabditis elegans*, Structure-activity relationship (SAR), Target identification, Thermal proteome profiling

## Abstract

Infections and diseases caused by parasitic nematodes have a major adverse impact on the health and productivity of animals and humans worldwide. The control of these parasites often relies heavily on the treatment with commercially available chemical compounds (anthelmintics). However, the excessive or uncontrolled use of these compounds in livestock animals has led to major challenges linked to drug resistance in nematodes. Therefore, there is a need to develop new anthelmintics with novel mechanism(s) of action. Recently, we identified a small molecule, designated UMW-9729, with nematocidal activity against the free-living model organism *Caenorhabditis elegans*. Here, we evaluated UMW-9729's potential as an anthelmintic in a structure-activity relationship (SAR) study in *C. elegans* and the highly pathogenic, blood-feeding *Haemonchus contortus* (barber's pole worm), and explored the compound-target relationship using thermal proteome profiling (TPP). First, we synthesised and tested 25 analogues of UMW-9729 for their nematocidal activity in both *H. contortus* (larvae and adults) and *C. elegans* (young adults), establishing a preliminary nematocidal pharmacophore for both species. We identified several compounds with marked activity against either *H. contortus* or *C. elegans* which had greater efficacy than UMW-9729, and found a significant divergence in compound bioactivity between these two nematode species. We also identified a UMW-9729 analogue, designated **25**, that moderately inhibited the motility of adult female *H. contortus in vitro*. Subsequently, we inferred three *H. contortus* proteins (HCON_00134350, HCON_00021470 and HCON_00099760) and five *C. elegans* proteins (F30A10.9, F15B9.8, B0361.6, DNC-4 and UNC-11) that interacted directly with UMW-9729; however, no conserved protein target was shared between the two nematode species. Future work aims to extend the SAR investigation in these and other parasitic nematode species, and validate individual proteins identified here as possible targets of UMW-9729. Overall, the present study evaluates this anthelmintic candidate and highlights some challenges associated with early anthelmintic investigation.

## Highlights

• A new chemical entity, UMW-9729, has anthelmintic efficacy on *H. contortus* and *C. elegans.*
• UMW-9729 and analogues display a significant bioactivity divergence across nematode species.
• One non-toxic analogue, **25**, shows moderated inhibition of *H. contortus* adult motility.
• Several *H. contortus* and *C. elegans* protein targets investigated *via* thermal proteome profiling.
• No conserved protein target suggests UMW-9729 has a unique antiparasitic mechanism of action.

## Graphical abstract

**Figure:**
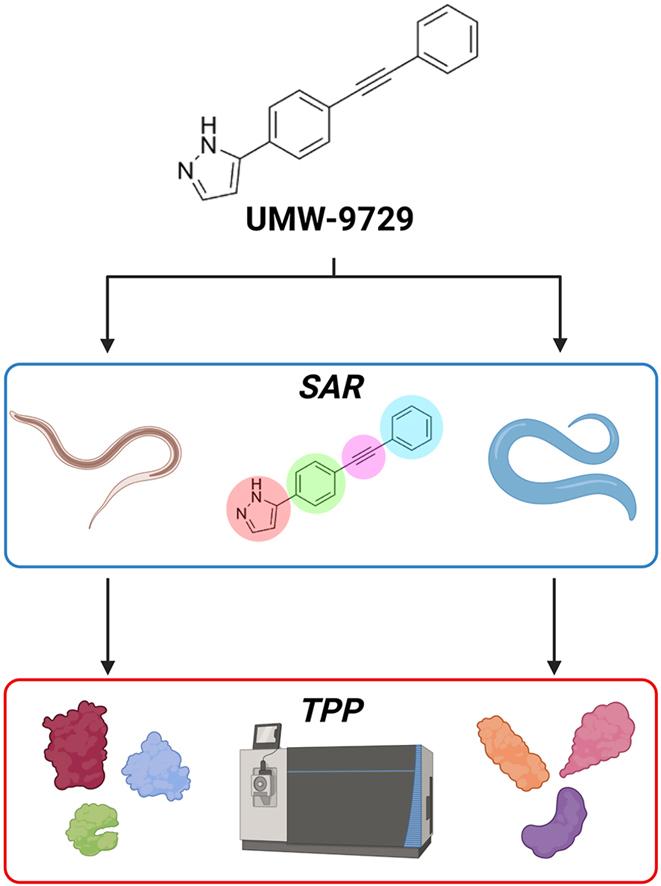

## 1 Introduction

Diseases caused by parasitic roundworms (nematodes) disproportionately affect billions of poverty-stricken people worldwide (Casuli, 2021; World Health Organization, 2022) and result in billions of dollars in losses to the global livestock industries (Charlier et al., 2021; Shephard et al., 2022). To combat this, integrated parasite control programs have been implemented to support both human (Tinkler, 2020; Montresor et al., 2020, 2022) and animal (Kahn and Woodgate, 2012; Terrill et al., 2012; Kearney et al., 2016; Maqbool et al., 2017) health, with chemotherapeutic (anthelmintic) treatment being a core component of control.

Despite the significant burden of parasitic helminths on health, the majority of anthelmintics for use in humans were first developed as veterinary anthelmintics (Woods et al., 2007; Nixon et al., 2020). The challenges associated with anthelmintic discovery and development are numerous and often hindered by significant economic barriers (reviewed by Nixon et al., 2020). Since 2000, only two drugs, namely monepantel (Kaminsky et al., 2008) and derquantel (Lee et al., 2002), have been commercialised for use in livestock animals; neither of these compounds has yet been approved for use in humans. Despite the relatively slow rate of commercial drug development over the past decade, anthelmintic treatment remains a core component of helminth control programs. The reliance on anthelmintics to treat and control helminth infections has led to the widespread development of drug resistance in parasitic nematodes. Although difficult to establish for parasites of humans (Vercruysse et al., 2011), concerns about resistance have been raised in relation to parasitic nematodes such as *Ascaris lumbricoides* (see Krücken et al., 2017; Furtado et al., 2019) and *Onchocerca volvulus* (see Osei-Atweneboana et al., 2011). In parasitic nematodes of livestock animals, resistance is widespread and well-documented (reviewed by Kotze and Prichard, 2016; Kotze and Hunt, 2023). For example, the highly pathogenic, blood-feeding nematode of small ruminants, *Haemonchus contortus* and many related nematodes have become resistant in many countries to every class of anthelmintic currently on the market – except for derquantel, available as a combination therapy with abamectin (Startect®). However, there is evidence of reduced efficacy (93.8 % efficacy) of Startect® against *H. contortus* in Merino sheep (Sales and Love, 2016; Lamb et al., 2017), although it is unclear whether this finding is related to macrocyclic lactone and/or derquantel resistance development. Thus, the widespread development of anthelmintic resistance, coupled with a relatively slow rate of drug discovery, lends impetus to the development of new anthelmintics with novel mechanisms of action.

**Fig. 1:**
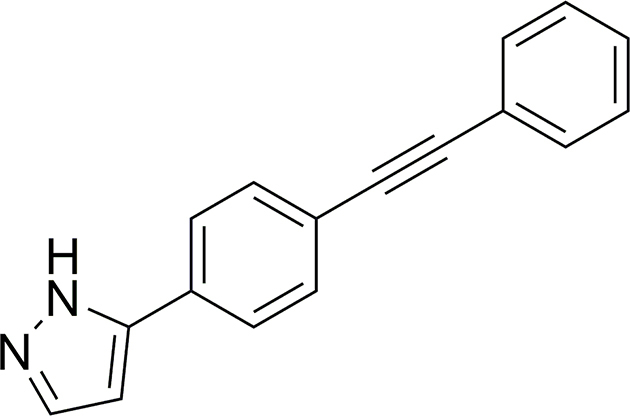
The chemical structure of UMW-9729.

Some recent anthelmintic drug discovery efforts (reviewed by Herath et al., 2022) have been centred around two nematodes – the strongylid *H. contortus* and the free-living *Caenorhabditis elegans*. Both of these species represent useful models for anthelmintic drug discovery, because they can be readily maintained and produced in a laboratory setting and are both related to numerous socioeconomically important nematodes (clade V; order Strongylida) of animals and humans. Moreover, the extensive availability of genomic, transcriptomic and proteomic resources and tools for these two species (Wang et al., 2019; Doyle et al., 2020; Davis et al., 2022) provides a solid basis for detailed investigations of the modes and mechanisms of action of currently-available and novel anthelmintic compounds. In previous work, an established high-throughput, whole-organism, motility-based phenotypic screening assay of the “HitFinder” library (*n* = 14,400; Maybridge; cf. Taki et al., 2021a) identified a compound, HF-00014, that had significant anthelmintic activity against *C. elegans*. HF-00014, herein referred to as UMW-9729 (Fig. 1), was shown to inhibit the motility of young adults of *C. elegans*, displaying a half-maximal inhibitory concentration (IC_5**0**_) of 5.6 μM (88.9 % maximum motility inhibition).

Although the available background information on UMW-9729 was limited, it was proposed that there was significant potential for further pre-clinical anthelmintic development. UMW-9729 (Fig. 1) is composed of three aryl ring systems and an alkyne linker. Further, it was proposed that the synthesis of a UMW-9729 analogue series was feasible, with the relatively simple structure providing an opportunity to produce several bioactive compounds. As such, UMW-9729 presented as a promising candidate for further medicinal chemistry optimisation as a nematocide *via* a quantitative structure-activity relationship and a drug target identification study. Here, we (i) synthesised a series of UMW-9729 analogues; (ii) assessed the anthelmintic activity of these analogues on both *H. contortus* and *C. elegans*; (iii) evaluated the cytotoxicity and mitotoxicity of selected analogues on HepG2 human hepatoma cells; and (iv) inferred potential protein targets of UMW-9729 in each *H. contortus* and *C. elegans*.

## 2 Materials and methods

### 2.1 Biological assays

#### 2.1.1 H. contortus larvae and adult procurement

*H. contortus* (Haecon-5 strain; cf. Schwarz et al., 2013) was produced in experimental sheep as described previously (Schwarz et al., 2013; Preston et al., 2015) and in accordance with the institutional animal ethics guidelines (permit no. 23983-2811-4; The University of Melbourne, Parkville, VIC, Australia). Helminth-free Merino sheep (six months of age; male) were orally inoculated with 7000 third-stage larvae (L3s) of *H. contortus*. Four weeks after inoculation, faecal samples were collected from sheep with patent *H. contortus* infection. These samples were incubated at 27 ^°^C and >90 % relative humidity for one week to yield L3s (Preston et al., 2015), which were then collected in tap water and allowed to migrate through two layers of nylon mesh (pore size: 20 μm; Rowe Scientific, Doveton, VIC, Australia) to remove debris. Clean L3s were stored in the dark at 11 ^°^C for up to six months (Preston et al., 2015).

Adult *H. contortus* were collected from the abomasa of sheep infected for 10 weeks, washed extensively with phosphate-buffered saline (PBS, pH 7.4) and subsequently in RPMI 1640 media supplemented with final concentrations of 2 mM L-glutamine, 100 IU/mL of penicillin, 100 μg/mL of streptomycin and 0.25 μg/mL of amphotericin B (Thermo Fisher Scientific, Scoresby, VIC, Australia; this supplemented RPMI was designated RPMI*). Female and male worms were collected and separated immediately prior to compound testing.

#### 2.1.2 H. contortus larvae preparation and dose-response assay

Immediately prior to use in assays, *H. contortus* L3s were exsheathed *via* exposure to 0.15 % (*v*/*v*) sodium hypochlorite for 20 min at 38 ^°^C (Preston et al., 2015), achieving an exsheathment rate of 90 %. The larvae were then immediately washed five times with 50 mL of sterile physiological saline (pH 7.0) by centrifugation at 2000×*g* (5 min) and resuspended (at a concentration of 300 xL3s per 50 μL) in sterile (autoclaved) lysogeny broth (LB; cf. Bertani, 1951; Taki et al., 2021b), supplemented with final concentrations of 100 IU/mL of penicillin, 100 μg/mL of streptomycin and 0.25 μg/mL of amphotericin B (Fungizone®, cat. No. 15240-062, Gibco, Thermo Fisher Scientific, Waltham, MA, USA); this supplemented LB was designated LB*.

The dose-response assay for *H. contortus* followed a well-established protocol (Taki et al., 2021b); it was employed to evaluate the potency of hit compounds against this nematode. Test compounds were assessed individually for an effect on the motility of xL3s (10-point, 2-fold serial dilution in LB*, 40 μM–0.16 μM). One compound, monepantel (prepared in the same manner as the test compounds), was used as a positive control. A solution of LB* was used as a negative control. The test compounds and positive control compounds (monepantel and moxidectin) were arrayed in triplicate across individual flat-bottom 96-well microplates, with six wells on each plate containing the negative control. Added to each well were 300 xL3s of *H. contortus* in 50 μL of LB* to give a final volume of 100 μL. Plates were then placed in a CO_2_ incubator (10 % (*v*/*v*) CO_2_, 38 ^°^C, >90 % humidity; Forma, model no. 311, Thermo Fisher Scientific, USA). After 168 h of incubation, worm motility was measured using a WMicroTracker ONE unit (PhylumTECH, Santa Fe, Argentina). Over a period of 15 min, disturbance of an infrared beam in individual wells was recorded as an ‘activity count’. Raw ‘activity counts’ for individual wells were normalised to the negative-controls. The compound concentrations were log_10_-transformed and fitted using a variable slope four-parameter equation, using the ordinary least squares fit model, employing Prism (v.9.1.0 GraphPad Software, San Diego, CA, USA). Larval development was established at 168 h of incubation with compound, as described previously (Preston et al., 2015). The phenotypes of larvae were examined using a microscope (Preston et al., 2015) and recorded.

#### 2.1.3 C. elegans preparation and dose-response assay

For the assay, *C. elegans* (N2 – wildtype Bristol strain) was maintained in the laboratory under standard conditions at 20 ^°^C on nematode growth media (NGM) agar plates, with *Escherichia coli* OP50 as a food source (Stiernagle, 2006). Gravid adult worms were collected from NGM plates, washed with sterile M9 buffer and then treated with a solution containing 0.4 % (*v*/*v*) sodium hypochlorite and 170 mM sodium hydroxide for 4–8 min at 22–24 °C (room temperature) to release eggs (Stiernagle, 2006; Porta-de-la-Riva et al., 2012). The eggs were then washed five times with 15 mL of sterile M9 buffer (centrifugation at 500×*g*, 2 min). After washing, the egg pellet was suspended in 8 mL of M9 buffer in a 15 mL tube and gently agitated for 24 h at 22–24 °C to produce first-stage larvae (L1s); 45 h prior to screening, synchronised *C. elegans* L1s were inoculated on to NGM plates containing 500 μL of *E. coli* OP50 (∼3000 larvae per plate) and allowed to develop to fourth-stage larvae (L4s) at 20 °C. L4s were collected from plates and washed twice with sterile M9 buffer by centrifugation (500×*g*, 2 min) to remove *E. coli* OP50, and then resuspended to a concentration of 100 larvae per 50 μL in sterile (autoclaved) LB*.

The dose-response assay for *C. elegans* followed a well-established protocol (Taki et al., 2021a) and was employed to evaluate the potency of hit compounds against this nematode. Test compounds were assessed individually for an effect on the motility of *C. elegans* (10-point, 2-fold serial dilution in LB*; from 40 μM to 0.16 μM) in the transition from the L4 to the young adult stage. Two compounds, monepantel (Zolvix™; Elanco, Australia) and moxidectin (Cydectin®; Virbac, France) were used as positive controls and prepared in the same manner as the test compounds. A solution of LB* +0.25 % (*v*/*v*) dimethylsulfoxide (DMSO) was used as a negative control. The test compounds and positive control compounds were arrayed in triplicate across individual flat-bottom 96-well microplates (cat. no. 3596; Corning, Corning, NY, USA), with six wells on each plate containing the negative control. Added to each well were 100 *C. elegans* in 50 μL of LB* to give a final volume of 100 μL. Plates were then placed in an incubator (Heratherm, model no. IMP180, Thermo Fisher Scientific, USA) at 20 ^°^C for 40 h. At 40 h, worm activity (i.e. motility) was measured using a WMicroTracker ONE unit (Phylumtech, Sunchales, Santa Fe, Argentina). Over 15 min, disturbance of an infrared beam in individual wells was recorded as an ‘activity count’. Raw ‘activity counts’ for each well were normalised to the negative controls. The compound concentrations were log_10_-transformed and fitted using a variable slope four-parameter equation, using the ordinary least squares fit model, employing the program Prism (v.9.1.0 GraphPad Software, San Diego, CA, USA).

#### 2.1.4 Assessment of the activity of selected compounds on *H. contortus* adults

The activity of UMW-9729 and six derivatives (**12**, **14**, **15, 16**, **18** and **25**) was assessed on adult female specimens of *H. contortus* in an established assay (Taki et al., 2020). The compound was added in triplicate to the wells of a 24-well plate (cat. no. 3524; Corning, USA) at a concentration of 40 μM in 500 μL of RPMI* (RPMI supplemented with final concentrations of 2 mM L-glutamine, 100 IU/mL of penicillin, 100 μg/mL of streptomycin and 0.25 μg/mL of amphotericin B; Thermo Fisher Scientific, Scoresby, VIC, Australia). Two positive-control compounds, monepantel and moxidectin, and a negative control containing 1 % (*v*/*v*) DMSO only, were included in triplicates on the same plate. Three adult females were added to each of the triplicate wells containing either the test compound or the controls and placed in a CO_2_ incubator (10 % (*v*/*v*) CO_2_, 40 ^°^C, >90 % relative humidity) for 1 day. A video recording (30 s) of each well was taken at 3 h, 6 h, 12 h and 24 h during the total incubation period to assess the reduction in worm motility, which was scored as 3 (“good”), 2 (“low”), 1 (“very low”) or 0 (“no movement”; cf. Taki et al., 2020). For each test or control compound, the motility scores for each of the triplicate wells were calculated, normalised with reference to the negative control (100 % motility) and recorded as a percentage.

#### 2.1.5 HepG2 viability assays

##### 2.1.5.1 Cytotoxicity evaluation

The cytotoxicity of UMW-9729 and six key derivatives (**12**, **14**, **15, 16**, **18** and **25**) on human hepatoma (HepG2) cells was evaluated as described previously (Gilson et al., 2017). In short, HepG2 cells were first cultured (in an incubator at 5 % (*v*/*v*) CO_2_, 37 ^°^C, >90 % humidity) in Dulbecco's modified Eagle's medium (DMEM) supplemented with 5 % foetal bovine serum (FBS). Test compounds were serially-diluted (10-points, 2-fold serial dilution, top concentration of 50 μM) in DMEM + 10 % FBS, adjusted to a 0.5 % DMSO concentration and arrayed across a 384-well plate. Bortezomib (10 μM) was used as a positive control; 0.5 % DMSO was used as a negative control. HepG2 cells (1 × 10^3^ cells per 50 μL of DMEM + 10 % FBS) were then seeded into wells of the assay plate; plates were the incubated (5 % (*v*/*v*) CO_2_, 37 ^°^C, >90 % humidity) for 48 h. Cell proliferation was subsequently determined using CellTiter-Glo (Promega) and normalised using the negative-controls to calculate as a percentage. All compounds were tested in duplicate. The half-maximal cytotoxic (CC_50_) values were calculated by the Dotmatics (v.5.3) and Spotfire (v.7.11.1) software using a nonlinear regression four-parameter fit analysis.

##### 2.1.5.2 Mitotoxicity evaluation

The mitotoxicity of UMW-9729 and six key derivatives (**12**, **14**, **15, 16**, **18** and **25**) on human hepatoma (HepG2) cells was evaluated as described previously using an established protocol (Swiss and Will, 2011; Kamalian et al., 2015; Śliwka et al., 2016). Test compounds were serially-diluted (7-points, 2-fold serial dilution, 50 μM top concentration) in DMEM (Thermo Fisher Scientific, USA) with GlutaMax™ supplemented with 25 mM D-galactose, 10 % heat-inactivated FBS, 100 IU/mL of penicillin, 100 μg/mL of streptomycin and 0.25 μg/mL of amphotericin B (denoted DMEM*). Monepantel and moxidectin (prepared in the same manner as the test compounds) were included as reference compounds. M-666 (10 M; Le et al., 2018) was used as a positive control; 0.25 % DMSO was used as a negative control. HepG2 cells were seeded into wells of a 96-well plate in 80 μL of DMEM* (at 1 × 10^5^ cells per well) and allowed to adhere for 16 h (5 % (*v*/*v*) CO_2_, 37 ^°^C, >90% humidity)prior to incubation with individual compounds, at a final volume of 100 μL. Cells were starved of serum (DMEM* without FBS) for 4 h prior to the incubation with compounds (Swiss and Will, 2011; Kamalian et al., 2015). Following 48 h of incubation with compounds, cell viability was determined by crystal violet staining (Śliwka et al., 2016). The absorbance (595 nm) of treated cells was normalised using the negative-controls to calculate the cell viability. All compounds and controls were tested in triplicate. To determine the half-maximal mitotoxic concentration (MC_50_) values, compound concentrations were log_10_-transformed, baseline-corrected using M-666, and fitted using a nonlinear regression four-parameter fit analysis using Prism v.9.1.0.

#### 2.1.6 Thermal proteome profiling (TPP)

Thermal proteome profiling was conducted using an established five-step protocol (Taki et al., 2022).

##### 2.1.6.1 Preparation of protein extracts from *H. contortus* and *C. elegans*

*H. contortus* (2,000,000 L3s) and *C. elegans* (500,000 young adults) were prepared as previously described, concentrated (separately) by centrifugation (2000×*g*, 5 min) and frozen at −80 ^°^C, following the removal of the supernatant. Subsequently, the frozen pellets were ground to a fine powder in liquid nitrogen using a mortar and pestle, each transferred to an individual 10 mL tube, suspended in 3 mL ice-cold phosphate-buffered saline (pH 7.0) containing 0.5 % (*v*/*v*) nonyl phenoxypolyethoxylethanol (NP-40) and lysed by gentle aspiration/expulsion using a 5 mL sterile syringe with a 22-gauge needle. Subsequently, the supernatant was collected from each suspension following centrifugation at 20,000×*g* for 20 min at 4 ^°^C. The protein concentration in both supernatants were measured using a BCA Protein Assay Kit (Thermo Fisher Scientific, USA), adjusted to 2 mg/mL, and both supernatants were divided into four 250 μL aliquots (each containing 500 μg protein).

##### 2.1.6.2 Incubation with compound (UMW-9729) and temperature profile

From each group of four 250 μL aliquots (containing either *H. contortus* or *C. elegans* proteins), two (i.e. test-samples) were each incubated with an equal volume of compound (UMW-9729 at 50 μM), and two control-samples with an equal volume of PBS (pH 7.0) for 30 min at 23 ^°^C. Each of the samples (containing 500 μL) were partitioned into 10 PCR tubes (50 μL each); individual pairs of test- and control-samples were simultaneously incubated in a thermal cycler (Applied Biosystems) at 10 distinct temperatures (37, 41, 44, 47, 50, 53, 56, 59, 63 and 67 ^°^C) for 3 min. Subsequently, all 80 tubes were centrifuged 20,000×*g* for 20 min at 4 ^°^C, and soluble proteins (i.e. from above the pellet) collected into fresh tubes (each containing 45 μL).

##### 2.1.6.3 In-solution digestion and isobaric stable isotope labelling of peptides

Proteins in aliquots (45 μL) of individual samples (n = 80) were denatured in 8 M urea for 30 min at 37 ^°^C and diluted to < 2 M urea using lysis buffer prior to processing for in-solution digestion (Ang et al., 2011). Samples were reduced with 10 mM Tris (2-carboxyethyl) phosphine, alkylated with 55 mM iodoacetamide, followed by digestion with trypsin (Promega) at 37 ^°^C for 16 h. The trypsin-treated samples were acidified with 1.0 % (*v*/*v*) formic acid (FA) and purified using Oasis HLB cartridges (Waters, USA); wash solvent, 0.1 % FA; elution solvent, 80 % acetonitrile (CH_3_CN) in 0.1% FA). Then, proteins were labelled with tandem mass tags (TMTs) (Zecha et al., 2019). In brief, desalted peptides were resuspended in 50 mM triethylammonium bicarbonate (pH 8.5) and labelled with isobaric compounds using TMT10plex isobaric label reagent (Thermo Fisher Scientific, USA) that was dissolved in 41 μL of anhydrous CH_3_CN. The TMT-peptide mixture was incubated for 1 h at 25 ^°^C with gentle shaking. Subsequently, 3.2 μL of 5 % (*w*/*v*) hydroxylamine was added to the mixture and incubated for 15 min at 25 ^°^C with gentle shaking to quench the reaction. Labelled peptides were combined accordingly and then desalted on Oasis HLB cartridges (using wash solvent, 0.1 % FA; elution solvent, 80 % CH_3_CN in 0.1 % FA). Each mixed peptide sample was separated into eight fractions using the high pH reversed-phase peptide fractionation kit (Pierce), according to the manufacturer's protocol. All fractions were freeze-dried prior to resuspension in aqueous 2 % (*w*/*v*) CH_3_CN and 0.05 % (*w*/*v*) trifluoroacetic acid (TFA) before LC-MS/MS analysis.

##### 2.1.6.4 LC-MS/MS analysis, and protein identification/annotation

LC-MS/MS was performed on the Exploris 480 Orbitrap mass spectrometer (Thermo Fisher Scientific, USA). The LC system was equipped with an Acclaim Pepmap nano-trap column (Dinoex-C18, 100 Å, 75 μm × 2 cm) and an Acclaim Pepmap RSLC analytical column (Dinoex-C18, 100 Å, 75 μm–50 cm). The tryptic peptides were injected into the enrichment column at an isocratic flow of 5 μL/min of 2 % (*v*/*v*) CH_3_CN containing 0.05% (*v*/*v*) TFA for 6 min, applied before the enrichment column was switched in-line with the analytical column. The eluents were 0.1 % (*v*/*v*) FA (solvent A) in water and 100 % (*v*/*v*) CH_3_CN in 0.1 % (*v*/*v*) FA (solvent B), both supplemented with 5 % DMSO. The gradient was at 300 nL/min from (i) 0–6 min, 3 % B; (ii) 6–7 min, 3–4 % B; (iii) 7–82 min, 4–25 % B; (iv) 82–86 min, 25–40 % B; (v) 86–87 min, 40–80 % B; (vi) 87–90 min, 80–3 % B; (vii) 90–90.1 min, 80–3 % B and equilibrated at 3 % B for 10 min before injecting the next sample. The Exploris 480 Orbitrap mass spectrometer was operated in the data-dependent mode, whereby full MS1 spectra were acquired in a positive mode, with spray voltage at 1.9 kV, source temperature at 275 ^°^C, MS1 at 120,000 resolution, normalised AGC target of 300 % and maximum IT time of 25 ms. The top 3 s method was used and selecting peptide ions with charge states of ≥ 2–7 and intensity thresholds of ≥5 × 10^−3^ were isolated for MS/MS. The isolation window was set at 0.7 *m/z*, and precursors were fragmented using higher energy C-trap dissociation (HCD) at a normalised collision energy of 35, a resolution of 30,000 (TurboTMT activated), a normalised AGC target of 200 % and automated IT time.

Mass spectrometry data were processed using MaxQuant (v2.1.1.0) for the identification and quantification of peptides/proteins. Proteins were matched to those inferred from the reference genome (version 4) for *H. contortus* (Doyle et al., 2020) or *C. elegans* (PRJNA13758). The TMT reagent was corrected for natural carbon isotopes and incomplete stable isotope incorporation. Fixed modifications of carbamidomethylation of cysteine. Trypsin/P was set as the protease with a maximum of 2 missed cleavages. Variable modifications are oxidation of methionine and acetylation of protein N-terminus. All quantitative values were normalised based on the weighted ratio to reference channel function to the 1st TMT reference channel (126C) made up of a pool of each sample. The isobaric matching between runs feature to improve reporter ion-based quantitation was also turned on. Protein and PSM false discovery rates (FDR) were both set at < 0.01. Results are available *via* the PRIDE data repository (accession number: PXD048945).

##### 2.1.6.5 Data processing and analysis

The quantitative protein data produced by MaxQuant was taken for analysis in R (v4.1.2). Decoy proteins, contaminant proteins, proteins only identified by modified peptides, and proteins that were identified by less than 2 razor or unique peptides were removed. Corrected reporter ion intensities were then divided by the intensity of the 37 ^°^C channel. Due to the marked decrease in overall protein abundance with increasing temperature, protein abundance ratios were grouped by treatment temperature and subjected to quantile normalisation using the software package limma (v3.50.0; Ritchie et al., 2015). Proteins were filtered to retain only those with non-zero values for each sample, and these were taken for subsequent analysis.

Thermal profiles of quantified proteins were assessed using the package NPARC (v1.6.0; Childs et al., 2019), which fits nonparametric models to the temperature profile data under null and alternative hypotheses; p-values were then calculated from F-statistics with empirically estimated degrees of freedom, as described in the NPARC package documentation (Perrin et al., 2020). Melting profiles were plotted and manually inspected for top ranking protein hits that were statistically significant (Benjamini-Hochberg-adjusted p-values were <0.01).

### 2.2 General chemistry experimental

All non-aqueous reactions were performed under an atmosphere of nitrogen, unless otherwise specified. Commercially available reagents were used without further purification. Flash chromatography was performed with silica gel 60 (particle size 0.040–0.063 μm) on a CombiFlash Rf Purification System (Teledyne Isco) with mobile phase gradients as specified. NMR spectra were recorded on a Bruker Avance DRX 300 with the solvents indicated (^q^H NMR at 300 MHz). Chemical shifts are reported in ppm on the δ scale and referenced to the appropriate solvent peak. Chemical shifts reported in ^19^F NMR are referenced to an external standard (trifluoroacetic acid) in the solvent indicated (Rosenau et al., 2018). LCMS were analysed on an Agilent LCMS system equipped with an Agilent G6120B Mass Detector, 1260 Infinity G1312B Binary pump, 1260 Infinity G1367E HiPALS autosampler, and 1260 Infinity G4212B Diode Array Detector. The LCMS conditions were as follows: column: Luna Omega (1.6 μm, C18, 50 × 2.1 mm); injection volume: 1 μL; gradient: 5–100 % B over 3.8 min (solvent A: water/0.1 % FA; solvent B: CH_3_CN/0.1 % FA); acquisition time: 4.1 min; flow rate: 1 mL/min; detection: 254 and 214 nm. Unless otherwise noted, all compounds were found to be >95 % pure by this method. HRMS was performed through the Bio21 Mass Spectrometry and Proteomics Facility and recorded on a Thermo Scientific nano-LC Q Exactive Plus Mass spectrometer with electrospray ionisation (ESI). Synthetic procedures and compound characterisations are in **Additional File 1**: **S5**; ^1^H,^13^C and ^19^F spectra, and HPLC traces, of UMW-9729 and all analogues are in **Additional File 1**: Fig. S6.

## 3 Results

### 3.1 Synthesis of analogues

To develop a structure-activity profile, a series of structural changes were made to define the anthelmintic activity of UMW-9729. The compound series was then assessed for potency in a dose-response assay on exsheathed third-stage larvae (xL3s) of *H. contortus* and young adults of *C. elegans*.

**Scheme 1:**
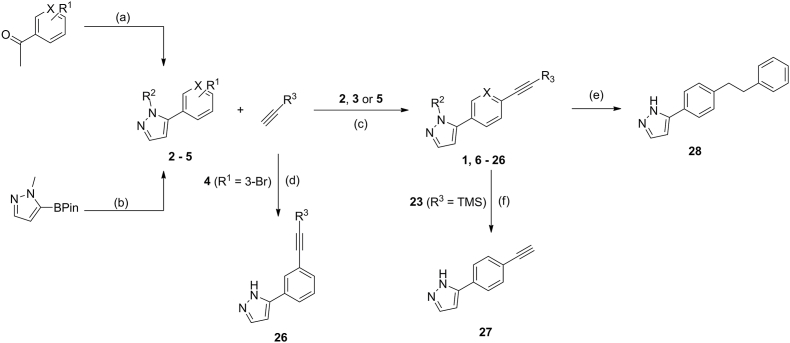
Synthetic pathway towards UMW-9729 and analogues. (a) (i) DMF-DMA, 80 ^°^C, 12 h (ii) hydrazine monohydrate, EtOH, 70 ^°^C, 3 h; (b) 1-bromo-4-iodobenzene, Pd(dppf)Cl_2_, K_2_CO_3_, 9:1 1,4-dioxane/water, 90 ^°^C, 12 h; (c) Pd(dppf)Cl_2_, CuI, DIPEA, DMF, 50 ^°^C, 24 h; (d) Pd(OAc)_2_, xantphos, K_3_PO_4_, toluene, 120 ^°^C, 72 h; (e) Pd/C, H_2_, 20 ^°^C, 6 h; (f) K_2_CO_3_, MeOH, 20 ^°^C, 6 h.

The synthetic route towards UMW-9729 (**1**) (and subsequent analogues) began with the treatment of 1-(4-iodophenyl)ethanone with *N,N*-dimethylformamide dimethyl acetal and hydrazine monohydrate, to form the intermediate compound 5-(4-iodophenyl)-1*H*-pyrazole (**2**) in 78 % yield (Scheme 1). Intermediate compounds **3** and **4** were synthesised in a similar fashion, utilising 1-(6-bromo-3-pyridyl)ethanone and 1-(3-bromophenyl)ethanone, respectively. Compound **5** was synthesised *via* a Suzuki coupling between 1-bromo-4-iodobenzene and 1-methyl-1*H*-pyrazole-5-boronic acid pinacol ester using catalytic Pd(dppf)Cl_2_. Compound **2** was subsequently coupled with ethynylbenzene under Sonogashira reaction conditions utilising Pd(PPh_3_)_2_Cl_2_ as a catalyst to synthesise UMW-9729 in 71 % yield (Scheme 1); the same pathway, using a number of unique ethynylbenzene derivatives, afforded compounds **6**–**23** in varying yields (21–84 %); compounds **24** and **25** were accessed under the same conditions *via* the coupling of intermediates **3** or **5** with ethynylbenzene, respectively. Notably, 5-(3-(phenylethynyl)phenyl)-1H-pyrazole (**26**) was accessed *via* a copper-free Sonogashira coupling reaction (Pd(OAc)_2_ and xantphos) between (**4**) and ethynylbenzene. Trimethylsilyl deprotection of compound **23** using K_2_CO_3_ in MeOH gave 5-(4-ethynylphenyl)-1*H*-pyrazole (**27**), whereas the alkyl compound **28** was accessed *via* Pd/C-mediated hydrogenation of UMW-9729.

**Scheme 2:**
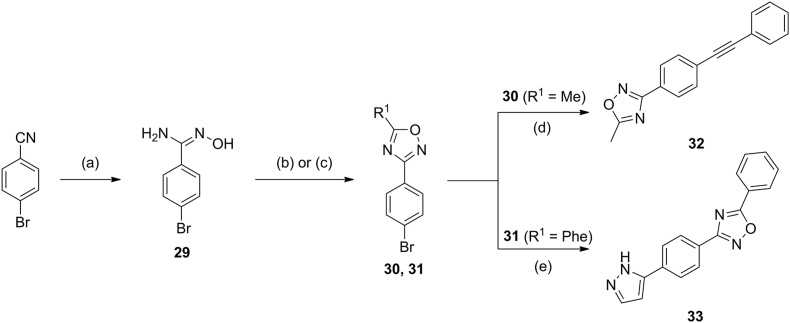
Synthetic pathway to access oxadiazole derivatives. (a) NH_4_OH, EtOH, 80 ^°^C, 8 h; (b) acetic acid, HATU, DIPEA, DMF, 20–100 ^°^C, 12 h; (c) benzoic acid, HATU, DIPEA, DMF, 20–100 ^°^C, 12 h; (d) ethynylbenzene, Pd(dppf)Cl_2_, CuI, DIPEA, DMF, 50 ^°^C, 24 h; (e) (1-(*tert*-butoxycarbonyl)-1*H*-pyrazol-5-yl)boronic acid, Pd(dppf)Cl_2_.DCM, K_2_CO_3_, 1,4-dioxane/water 9:1, 90 ^°^C, 24 h (note, the *boc* protecting group was inadvertently removed under these conditions).

The synthesis of the oxadiazole derivatives began with generating 4-bromo-*N*-hydroxybenzimidamide (**29**) *via* the treatment of 4-bromobenzonitrile with NH_4_OH (Scheme 2). HATU-mediated cyclisation of **29** with either acetic acid or benzoic acid gave intermediate compounds **30** and **31**, respectively. **30** was coupled with ethynylbenzene under Sonogashira conditions to give compound **32**; **31** was coupled with (1-(*tert*-butoxycarbonyl)-1*H-*pyrazol-5-yl)boronic acid under Suzuki conditions to give compound **33** – of note, the *boc*-protecting group was also removed under these reaction conditions.

### 3.2 Dose-response assessment of UMW-9729 for nematocidal activity against H. contortus

#### 3.2.1 Alterations to the terminal phenyl ring identify an analogue with greater potency

UMW-9729 was first assessed for its ability to reduce motility in xL3s of *H. contortus*, displaying an IC_50_ of 6.7 μM (maximum motility inhibition, MMI = 84 %; **Additional File 1:** Fig. S1). Comparatively, monepantel and moxidectin displayed IC_50_ values of 0.6 μM (MMI = 96 %) and 18 μM (MMI = 77 %), respectively. Alterations to the terminal aryl ring began with exploring 2-position substituents (Table 1). Here, a 2-OMe (**6**) or -CF_3_ (**7**) addition resulted in a complete loss of activity (>40 μM). A -F substitution (**8**) displayed moderately decreased activity (IC_50_ of 12 μM, MMI = 98 %), whereas a bulkier -Cl substitution (**9**) retained some activity (IC_50_ = 4.8 μM), yet had a decreased MMI (62 %). Incorporating a 2-pyridine moiety (**10**) also resulted in a loss in activity (IC_50_ of 8.7 μM, MMI = 54 %).

**Table 1:**
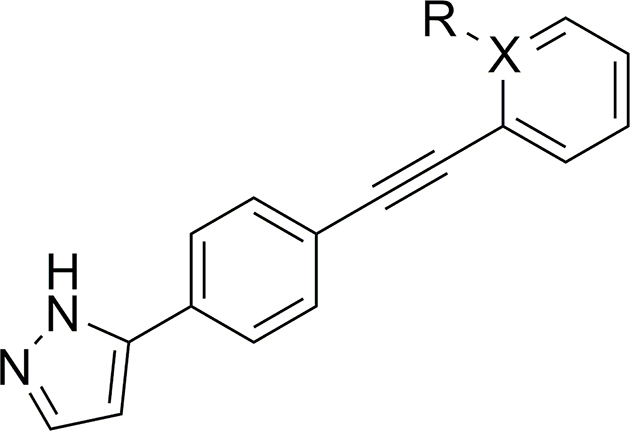
Activity of 2-substituted benzene UMW-9729 analogues on larvae of *H. contortus* (90 h incubation) and *C. elegans* (40 h incubation).

Compound	R	X^a^	Worm motility IC_50_ ± SD, μM^b^^,^^c^	
			*H. contortus* xL3s	*C. elegans* young adults
**UMW-9729**	H	–	6.7 ± 0.4 (84)	14 ± 2.9 (85)
**6**	OMe	–	>40	>40
**7**	CF_3_	–	>40	>40
**8**	F	–	13 ± 4.5 (98)	3.7 ± 1.9 (67)
**9**	Cl	–	4.8 ± 0.7 (62)	>40
**10**	–	N	8.7 ± 2.5 (54)	>40
Monepantel	N/A	N/A	0.6 ± 0.2 (96)	0.03 ± 0.01 (93)
Moxidectin	N/A	N/A	18 ± 9.1 (77)	0.003 ± 0.01 (100)

a Dash ‘– ‘indicates that X = ‘C’.

b IC_50_ calculated from three independent assays in triplicate.

c (Maximum motility inhibition, %).

At the 3-position of the aryl ring (Table 2), incorporation of a -Cl (**15**) or -OCF_3_ (**13**) group resulted in a complete loss of activity (>40 μM), whereas a 3-OH derivative (**17**) showed greatly reduced activity (29 μM, MMI of 54 %). The addition of a -Me group (**11**) displayed slightly lower activity (13 μM), whereas incorporation of -CF_3_ (**12**) or -F (**14**) retained activity equipotent activity (8.2 and 5.2 μM respectively; **Additional File 1:** Fig. S1), relative to UMW-9729. This retention of the potency of both **12** and **14**, compared to the loss in activity in **13** and **15**, indicated that small, electron-withdrawing groups may be preferred at the 3 position. Finally, potency assessment of a 3-pyridine derivative (**16**) was found to be equipotent in *H. contortus* – possibly providing a pathway for future inclusion of polar groups (**Additional File 1:** Fig. S1).

**Table 2:**
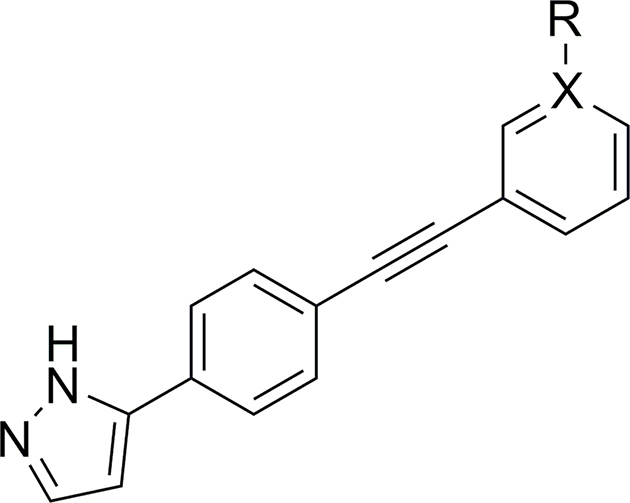
Activity of 3-substituted benzene UMW-9729 analogues on larvae of *H. contortus* (90 h incubation) and *C. elegans* (40 h incubation).

Compound	R	X^a^	Worm motility IC_50_ ± SD, μM^b^^,^^c^	
			*H. contortus* xL3s	*C. elegans* young adults
**UMW-9729**	H	–	6.7 ± 0.4 (84)	14 ± 2.9 (85)
**11**	Me	–	13 ± 5.7 (90)	>40
**12**	CF_3_	–	8.2 ± 1.9 (80)	12 ± 7 (75)
**13**	OCF_3_	–	>40	>40
**14**	F	–	5.2 ± 0.9 (100)	8.0 ± 1.8 (87)
**15**	Cl	–	>40	8.2 ± 7.8 (90)
**16**	–	N	6.8 ± 0.1 (94)	>40
**17**	OH	–	29 ± 12 (54)	25 ± 7 (71)
Monepantel	N/A	N/A	0.6 ± 0.2 (96)	0.03 ± 0.01 (93)
Moxidectin	N/A	N/A	18 ± 9.1 (77)	0.003 ± 0.01 (100)

a Dash ‘– ‘indicates that X = ‘C’.

b IC_50_ calculated from three independent assays in triplicate.

c (Maximum motility inhibition, %).

At the 4-position of the terminal aryl ring (Table 3), the -OMe (**19**) and the 4-pyridine (**22**) derivatives were inactive. Incorporations of a 4-Cl (**20**) or 4-CN (**21**) functional group displayed similar IC_50_'s to original compound (3.7 and 6.7 μM IC_50_ respectively) yet had lower MMI's (56 and 67 % respectively). Finally, analogue **18**, containing a 4-Me substitution, was found to have greater potency than UMW-9729, displaying an IC_50_ of 2.0 μM (MMI = 89 %) against *H. contortus* larvae (**Additional File 1**: Fig. S1).

**Table 3:**
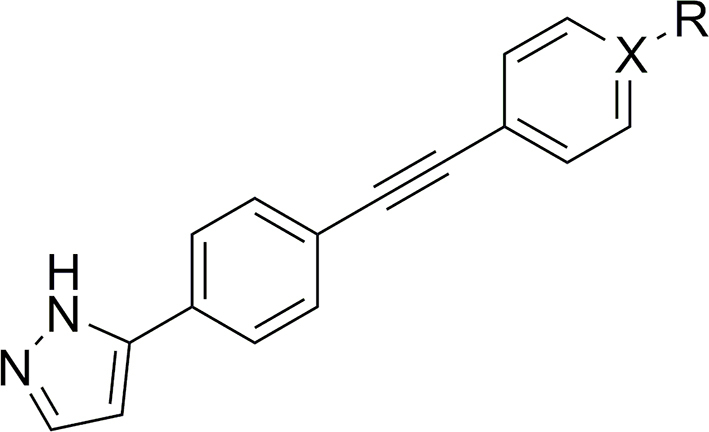
Activity of 4-substituted benzene UMW-9729 analogues on larvae of *H. contortus* (90 h incubation) and *C. elegans* (40 h incubation).

Compound	R	X^a^	Worm motility IC_50_ ± SD, μM^b^^,^^c^	
			*H. contortus* xL3s	*C. elegans* young adults
**UMW-9729**	H	–	6.7 ± 0.4 (84)	14 ± 2.9 (85)
**18**	Me	–	2.0 ± 0.1 (89)	>40
**19**	OMe	–	>40	>40
**20**	Cl	–	3.7 ± 1.1 (56)	>40
**21**	CN	–	6.7 ± 4.2 (67)	>40
**22**	–	N	>40	>40
Monepantel	N/A	N/A	0.6 ± 0.2 (96)	0.03 ± 0.01 (93)
Moxidectin	N/A	N/A	18 ± 9.1 (77)	0.003 ± 0.01 (100)

a Dash ‘– ‘indicates that X = ‘C’.

b IC_50_ calculated from three independent assays in triplicate.

c (Maximum motility inhibition, %).

#### 3.2.2 N-methylation of pyrazole motif associated with enhanced potency

*N*-Methylation of the pyrazole motif (**25**, Table 4) gave a compound with an enhanced IC_50_ of 1.9 μM (MMI = 73 %; **Additional File 1:** Fig. S1). Furthermore, replacement of the pyrazole with a 5-methyl-1,2,4-oxadiazole moiety (**32**, IC_50_ = 5.1 μM, MMI of 76 %) gave a compound with similar potency to UMW-9729.

**Table 4:**
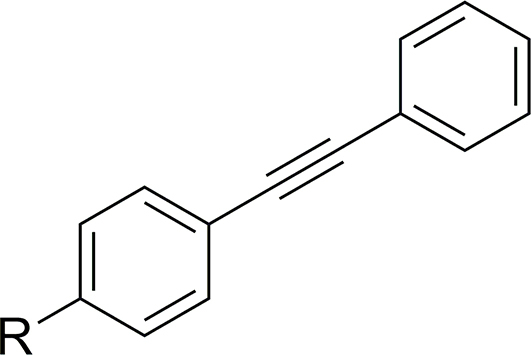
Activity of alkyne-substituted UMW-9729 analogues on larvae of *H. contortus* (90 h incubation) and *C. elegans* (40 h incubation).

Compound	R	Worm motility IC_50_ ± SD, μM^a^^,^^b^	
		*H. contortus* xL3s	*C. elegans* young adults
**UMW-9729**		6.7 ± 0.4 (84)	14 ± 2.9 (85)
**25**		1.9 ± 0.14 (73)	>40
**32**		5.1 ± 0.5 (76)	18 ± 11 (78)
Monepantel	N/A	0.6 ± 0.2 (96)	0.03 ± 0.01 (93)
Moxidectin	N/A	18 ± 9.1 (77)	0.003 ± 0.01 (100)

a IC_50_ calculated from three independent assays in triplicate.

b (Maximum motility inhibition, %).

#### 3.2.3 Changes to alkyne linker resulted in a loss of activity

Changing the point of attachment of the phenylacetylene motif (**26**) (Table 5) resulted in a complete loss of activity – similarly, reduction of the alkyne linker (Table 5) to its alkyl counterpart (**28**, IC_50_ = 16 μM, MMI of 57 %) or replacement with an oxadiazole functional group (**33**, IC_50_ = 2.5 μM, MMI of 55 %) resulted in a significant reduction of activity. Finally, replacement of the terminal phenyl with a trimethyl silyl group (**23**), or removal of the terminal phenyl ring (**27**), also resulted in a complete loss of activity. Attempts to incorporate a pyridine moiety within the central phenyl ring (**24**) also reduced activity (IC_50_ = 24 μM, MMI of 70 %).

**Table 5:**
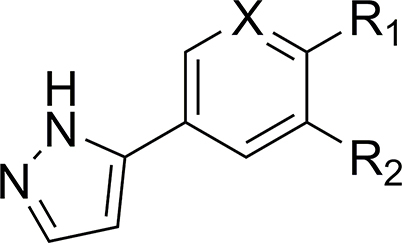
Activity of pyrazole-substituted UMW-9729 analogues on larvae of *H. contortus* (90 h incubation) and *C. elegans* (40 h incubation).

Compound	R_1_^a^	R_2_^a^	X^b^	Worm motility IC_50_ ± SD (μM)^c^^,^^d^	
				*H. contortus* xL3s	*C. elegans* young adults
**UMW-9729**	Ethynylbenzene	–	–	6.7 ± 0.4 (84)	14 ± 2.9 (85)
**23**	Ethynyltrimethylsilane	–	–	>40	>40
**24**	Ethynylbenzene	–	N	24 ± 8.5 (70)	>40
**26**	–	Ethynylbenzene	–	>40	>40
**27**	Ethyne	–	–	>40	>40
**28**	Ethylbenzene	–	–	16 ± 4.9 (57)	>40
**33**		–	–	2.5 ± 2.7 (55)	>40
Monepantel	N/A	N/A	N/A	0.6 ± 0.2 (96)	0.03 ± 0.01 (93)
Moxidectin	N/A	N/A	N/A	18 ± 9.1 (77)	0.003 ± 0.01 (100)

a Dash ‘– ‘indicates that R_1_/R_2_ = ‘H’.

b Dash ‘– ‘indicates that X = ‘C-H’.

c IC_50_ calculated from three independent assays in triplicate.

d (Maximum motility inhibition, %).

### *3.3* *Dose-response assessment of* UMW-9729 *for nematocidal activity against C. elegans*

#### 3.3.1 Substituent addition to terminal phenyl ring overall linked to activity loss

The panel of compounds which were tested for activity on larvae of *H. contortus* were also tested for inhibitory effects on the motility of young adults of *C. elegans*. Here, UMW-9729 displayed an IC_50_ of 14 μM, reaching an MMI of 85 % (**Additional File 1**: Fig. S2). Comparatively, monepantel and moxidectin displayed IC_50_ values of 0.03 μM (MMI = 93 %) and 0.003 μM (MMI = 100 %), respectively.

Addition of a -OMe (**6**), -CF_3_ (**7**) or -Cl (**9**) at the 2-position of the terminal phenyl ring (Table 1) led to a complete loss of activity (>40 μM). Moreover, a 2-F substituted derivative (**8**) displayed a greater IC_50_ (3.7 μM) compared to the parent compound, but reached an MMI of 67 %. Finally, incorporation of a 2-pyridine (**10**) motif resulted in a loss of compound activity.

Changes to the 3-position of the terminal phenyl ring (Table 2) were generally well-tolerated compared to the 2-position. Incorporation of either a -Cl (**15**) or -F (**14**) functional group resulted in compounds with enhanced activities (IC_50_ values of 8.2 and 9.0 μM, respectively; **Additional File 1**: Fig. S2). A 3-CF_3_ derivative (**12**) was equipotent to UMW-9729, displaying an activity of 12 μM (75 % MMI; **Additional File 1**: Fig. S2) – in contrast, a -Me derivative (**11**) lost activity, suggesting that electron-withdrawing, lipophilic functional groups are preferred at this position. Finally, the incorporation of a 3-pyridine motif (**16**) resulted in a loss of activity; however, the inclusion of a 3-hydroxy (**17**) only slightly reduced activity (25 μM IC_50_), suggesting that the incorporation of hydrophilic functional groups at the 3-position may be possible. Changes at the 4-position (-Me (**18**), -OMe (**19**), -Cl (**20**), -CN (**21**), and 4-pyridine (**22**; Table 3) all led to a loss of motility inhibition in *C. elegans* (>40 μM).

#### 3.3.2 N-methylation of pyrazole group loses activity

An *N*-methylated variant of the pyrazole motif (**25**, Table 4) showed a loss in activity (>40 μM) while in comparison, the 5-methyl-1,2,4-oxadiazole derivative (**32**, IC_50_ = 18 μM, MMI of 78 %) was found to be equipotent to the original UMW-9729 compound.

#### 3.3.3 Replacement of alkyne and removal of terminal phenyl loses activity

Changing the phenyl acetylene point of attachment (**26**), removal of the terminal phenyl ring (**27**), reduction of the alkyne (**28**) or replacement of the alkyne with an oxadiazole motif (**33**) resulted in a loss of activity (>40 μM), indicating that the rigidity of UMW-9729 provided by the alkyne group is pivotal to the compound's nematocidal activity in *C. elegans*. Incorporation of a nitrogen within the central phenyl ring (**24**) caused a loss in activity.

### 3.4 One non-cytotoxic and non-mitotoxic analogue moderately inhibits adult female motility

**Fig. 2:**
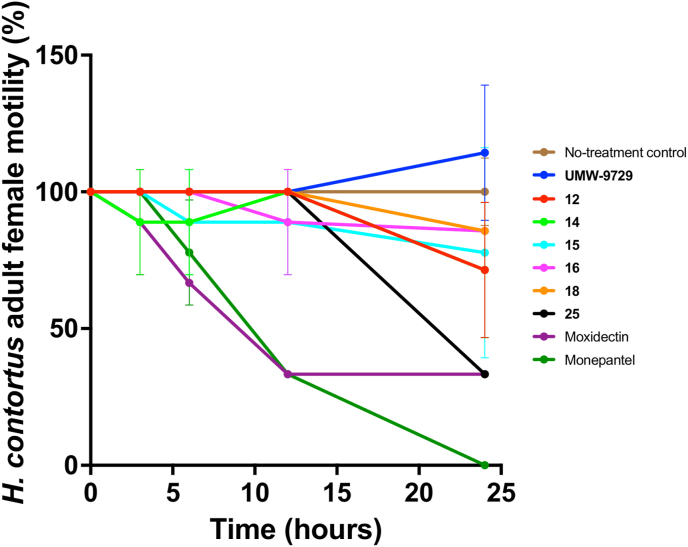
The *in vitro* motility inhibition (%) of UMW-9729 and six active derivative compounds (**12**, **14**, **15**, **16**, **18** and **25**) against adult females of *Haemonchus contortus*, with reference to two control compounds (monepantel and moxidectin). Motility scores (assessed at 3-, 6-, 12- and 24-h time points) for each compound were calculated and normalised to a negative control (100 % motility), and were recorded as a percentage. Data points represent one experiment conducted in triplicate; the mean ± standard deviation (SD).

We further assessed UMW-9729 and six key derivatives (**12**, **14**, **15, 16**, **18** and **25**) at a 40 μM concentration for the inhibition of motility in adult females of *H. contortus* after 24 h of incubation (Fig. 2). At 24 h, compounds **12** (29 %), **14** (29 %), **15** (23 %), **16** (15 %) and **18** (15 %) displayed a limited activity reduction, whereas UMW-9729 did not. Of note, compound **25** did not display a reduction in motility at 3 h, 6 h and 12 h time points, but reduced worm motility by 66 % after 24 h.

UMW-9729 and the six key derivatives were also assessed for cyto-toxic (*via* CellTiter-Glo) and mito-toxic (*via* crystal violet staining) effects on HepG2 human cells; all compounds were identified as non-cytotoxic and non-mitotoxic (half-maximal cytotoxic and mitotoxic concentrations: >40 μM; (**Additional File 1**: Fig. S3; **Additional File 1**: Fig. S4).

### 3.5 Proteomic investigation to infer targets in H. contortus and C. elegans

To investigate the possible protein targets of UMW-9729 in the nematode models, protein lysates of xL3s of *H. contortus* and L4s of *C. elegans* were individually incubated with 50 μM of UMW-9729 a then subjected to TPP across a gradient of 37 ^°^C–67 ^°^C, to identify proteins which are stabilised in the presence of UMW-9729. Using this technique, we first identified and quantified 4122 *H. contortus* proteins. Utilising a non-parametric analysis of the response curves (NPARC v.1.6.0; Childs et al., 2019), we assessed the thermal profiles of individual proteins and yielded 3270 melting profiles (**Additional File 2:** Table S3). Statistically significant protein target candidates (Benjamini-Hochberg adjusted p-values (pAdj) < 0.01) were then plotted and manually inspected; three proteins, designated HCON_00134350, HCON_00021470 and HCON_00099760, were stabilised in the presence of UMW-9729 (**Additional File 1**: Table S1).

Using this workflow, we also identified 5800 *C. elegans* proteins and 4700 distinct melting profiles (**Additional File 2:** Table S4); 14 of these proteins (designated F30A10.9, F15B9.8, PINN-4, UBL-5, D1086.10, PGP-1, H14N18.4, B0361.6, DNC-4, UNC-11, GST-15, ZNF-598, C01G6.4 and TRXR-1) stabilised in the presence of UMW-9729 (**Additional File 1**: Table S2). Furthermore, a literature search utilising the Online Gene Essentiality database (OGEE v3; Gurumayum et al., 2020) identified five of these proteins (F30A10.9, F15B9.8, SPOT-1, DNC-4 and UNC-11) as ‘conditional essential’ (cf. Campos et al., 2020; Gurumayum et al., 2020); the remaining proteins were categorised as ‘non-essential’.

As such, three *H. contortus* proteins (HCON_00134350, HCON_00021470 and HCON_00099760) and five *C. elegans* proteins (F30A10.9, F15B9.8, B0361.6, DNC-4 and UNC-11) were identified as possible candidates for further investigation as protein targets of UMW-9729.

## 4 Discussion

Here, we demonstrated that UMW-9729 displayed moderate anthelmintic activity against larvae of the highly pathogenic model nematode, *H. contortus*, and identified two derivatives, **18** and **25**, with a 3-fold increased activity when compared to the parent molecule. Further, we highlighted some key structural features contributing to the inhibition of worm motility. In brief, we found that, with the exception of an *ortho* -F or a *para* -Me functional group, additions to the terminal phenyl ring at either the *ortho* or *para* position were not tolerated. At the *meta* position, -Me, -CF_3_ and -F additions resulted in equipotent derivatives – however, inclusion of a bulkier -OCF_3_ or -Cl group resulted in a loss of activity. These differences suggest that the terminal phenyl may be oriented towards a binding cavity to accommodate the terminal aryl ring; however, the increased activity shown for the *para* -Me compound **18** suggests that there is space to extend further into this pocket. Moreover, *N-*methylation of the pyrazole motif on the UMW-9729 scaffold identified a compound (**25**) with greater activity, whereas a scaffold-hop to an oxadiazole moiety retained potency. Finally, efforts to replace the alkyne linker with a less rigid alkyl group, or an oxadiazole isostere, were not favourable towards activity. Of note, although no analogue synthesised here was more potent than monepantel, several analogues, including UMW-9729, were ∼3-fold more active than moxidectin. Additionally, the activity of moxidectin was relatively moderate, considering the reported anthelmintic *in vivo* field efficacy. We also tested UMW-9729 and several key derivatives (**12**, **14**, **15, 16**, **18** and **25**) for nematocidal effects against adult females of *H. contortus*; however, only one compound, **25**, displayed a moderate motility inhibition after 24 h of incubation. Thus, the apparent low effect of UMW-9729 against this parasite's most pathogenic stage (i.e. adult) of this parasite does suggest that the development of this compound as an effective anthelmintic may be limited.

The activities of UMW-9729 and its derivatives were also explored against *C. elegans*. Although we validated UMW-9729 as a moderate inhibitor of *C. elegans* motility, in general, structural changes were not well tolerated. On the terminal phenyl ring of UMW-9729, substitutions at the *ortho* or *para* position lost activity against this worm species, whereas only electron-withdrawing groups (such as -F, -Cl or -CF_3_) at the *meta* position demonstrated equipotent activity. Moreover, *N-*methylation of the pyrazole moiety gave a loss of activity, yet an oxadiazole motif was equipotent. Finally, changes to the alkyne linker, removal of the terminal phenyl ring or replacement with an oxadiazole motif also resulted in a loss of activity. Moreover, all analogues tested, including UMW-9729, were substantially less active against *C. elegans* than monepantel or moxidectin.

Several key differences in the activity of analogues between both *H. contortus* and *C. elegans* suggest that UMW-9729 may target two or more structurally distinct proteins in both nematode species. For instance, although the *para*-methyl derivative **18** was 3-fold more active than UMW-9729 in *H. contortus*, interestingly, this derivative was inactive against *C. elegans*. Another *N-*methylated pyrazole derivative, **25**, was also inactive against *C. elegans*, yet 3-fold more active against *H. contortus*. Conversely, a *meta*-Cl substitution on the terminal phenyl ring produced an analogue (**15**) with ∼ 2-fold increased activity than UMW-9729 against the free-living nematode species, contrasting a loss of activity against the parasitic worm. These differences in activity might be explained by the biological differences between *C. elegans* (free-living) and *H. contortus* (parasitic). In a future study, it would be of interest to assess the present collection of UMW-9729 analogues against other, closely related parasitic nematode species to identify whether there is a shared pharmacophore among these parasitic organisms.

To understand the mechanism of action responsible for the anthelmintic activity of UMW-9729, we used TPP (Savitski et al., 2014; Mateus et al., 2020; Taki et al., 2022) to identify UMW-9729-bound proteins in a lysate of *H. contortus* larvae. Here, we identified and prioritised three *H. contortus* proteins (named HCON_00134350, HCON_00021470 and HCON_00099760) which were significantly stabilised in the presence of UMW-9729. In each case, the function of the protein was inferred from the primary amino acid sequence (Doyle et al., 2020) and from the related *C. elegans* orthologue; in short, HCON_00134350 (*C. elegans* orthologue GLB-1, 54.9 % sequence identity, E-value of 6.8 × 10^−51^; Tilleman et al., 2011) was predicted to be a globin domain-containing protein whose function is associated with heme-binding; HCON_00021470 (*C. elegans* orthologue CDC-5L, 81.2% identity, E-value of 0; Shiimori et al., 2013) was predicted to be a cell division cycle 5-like protein whose function is associated with mRNA splicing; HCON_00099760 (*C. elegans* orthologue LPR-2, 66.7 % identity, E-value of 1.3 × 10^−114^; Forman-Rubinsky et al., 2017) was predicted to be apolipoprotein D whose function is linked to retinoid binding activity. Although the functions of the *H. contortus* proteins identified here have not yet been fully established, it is possible that the disruption of one or more of these proteins leads to worm immobilisation.

To explore whether UMW-9729 interacted with a conserved nematode protein target, we also used TPP to identify proteins which bind to UMW-9729 in a *C. elegans* lysate. Here, we identified five structures (named F30A10.9, F15B9.8, SPOT-1, DNC-4 and UNC-11) which were stabilised in the presence of UMW-9729 and recognised as ‘conditionally essential’ (OGEE v3; Gurumayum et al., 2020). The functional annotations for individual proteins, accessed *via* WormBase (https://wormbase.org//#012-34-5; Harris et al., 2020), predicted that F30A10.9 is involved in nuclear ribosomal RNA processing (human orthologue UTP24, cf. Wells et al., 2016); F15B9.8 is a predicted thrombospondin-type protein; SPOT-1 enables methyltransferase activity (human orthologue C9orf114/SPOUT01, cf. Treiber et al., 2017); DNC-4 is part of the dynactin complex (O'Rourke et al., 2011); UNC-11 enables SNARE binding (Nonet et al., 1999).

Notably, none of the proteins predicted here as targets of UMW-9729 were shared by *C. elegans* and *H. contortus*. In concert with the SAR investigation, this finding suggests that UMW-9729 does not share a protein target in both species. It is possible that the functional processes altered/interrupted by UMW-9729 in the parasitic nematode are not present in the free-living *C. elegans* worm (cf. Geary and Thompson, 2001) and, hence, UMW-9729 achieves anthelmintic activity *via* divergent pathways. Although these results may question the use of *C. elegans* as a surrogate model for antiparasitic discovery, it is clear, through the development of monepantel (Kaminsky et al., 2008) and the anthelmintic candidate Nemacol (Harrington et al., 2023), that *C. elegans* remains a useful system, with the caveat that drug testing also needs to be undertaken against one or more pertinent parasitic nematodes, including *H. contortus*.

An alternative explanation is that, in the case of UMW-9729, TPP may not be adequate to unequivocally define the target(s) of this compound. Possibly, orthogonal approaches, aimed at validating the protein targets identified here, could illuminate the genuine mode(s) of action of UMW-9729 in a nematode model. Complementary protein-focussed investigations, such as isothermal dose-response fingerprinting (Jafari et al., 2014) or affinity-based assays (Him et al., 2009; Seo and Corson, 2019), or genomics-directed studies, such as RNA interference (Blanchard et al., 2018; Hou et al., 2023), CRISPR/Cas9 genome editing (cf. Waaijers et al., 2013; Quinzo et al., 2022) or resistance-based studies utilising either *H. contortus* (see Kaminsky et al., 2008) or *C. elegans* (see Burns et al., 2006), could be employed to identify and/or validate drug-protein interactions. Of note, the binding mode of a structurally similar aryl alkyne compound, designated CHIR-090, in complex with a gram-negative bacteria specific protein has been previously elucidated (Brown et al., 2012). Although (presumably) CHIR-090 and UMW-9729 do not share the same target, the binding pocket interactions could be similar given the shared 1,2-diphenylethyne chemical moiety, and the interactions identified there (Brown et al., 2012) could assist future mechanism of action studies. Similarly, *in silico* methods (Trott and Olson, 2010) could also be used to generate hypotheses as to how UMW-9729 and its derivatives interact with identified proteins, to understand whether predicted interactions are reflected in the SAR results.

Given the divergence in the nematocidal pharmacophore between *H. contortus* and *C. elegans* worms, the low-to-moderate activity against *H. contortus* adult worms, and the apparent lack of a conserved nematode drug target, the future development of UMW-9729 as a broad-spectrum anthelmintic may be challenging. Certainly, future work should focus on the development of a non-cytotoxic and non-mitotoxic compound (with adequate pharmacokinetic properties) which is active against parasitic stages of *H. contortus* and other socioeconomically important nematodes (Keiser et al., 2016; Keiser and Häberli, 2021). Moreover, if a UMW-9729 analogue were established as a suitable front-runner candidate *in vitro*, it would be pivotal to also assess its antiparasitic activity *in vivo*. Finally, the validation of the protein targets inferred here, through complementary and/or orthogonal approaches, would be critical for the development of a UMW-9729-derived anthelmintic compound with a novel mechanism of action.

## Ethics approval

This study was conducted in accordance with the institutional animal ethics guidelines (permit no. 23983-2811-4; The University of Melbourne).

## Availability of data and materials

All data generated or analysed during this study are included in this published article and its supplementary information files. The datasets presented in this study have been deposited in the PRIDE repository with the accession number PXD048945.

## Funding

We gratefully acknowledge financial support from the 10.13039/501100000923Australian Research Council (LP220200614 and LP180101085), PhylumTECH and Oz Omics Pty Ltd.

## Authors’ contributions

RBG, BES and HTS formulated the overarching research goals. ACT, TW, JJB, NN and HTS developed the methodology; ACT, TW, NN, C-SA, MGL, and HTS conducted the research; ACT, MGL and HTS analysed the data; ACT and HTS validated the results. HTS and RBG drafted the manuscript, with valuable revisions conducted by BES, ACT, NN, TW, JJB, C-SA, MGL, NW, BCHC and AJ. This project was supervised and administrated by RBG, BES, ACT and NN. RBG, AJ, BCHC and BES acquired the funding for this project. All authors have read and agreed to the published version of the manuscript.

## Declaration of competing interest

The authors declare that they have no known competing financial interests or personal relationships that could have appeared to influence the work reported in this paper.
