# Supplementary material for: Comparative structure activity and target exploration of 1,2-diphenylethynes in Haemonchus contortus and Caenorhabditis elegans
Source: Int J Parasitol Drugs Drug Resist. 2024 Mar 19;25:100534. doi: 10.1016/j.ijpddr.2024.100534 (PMC10992699; doi:10.1016/j.ijpddr.2024.100534)
Supplement: Multimedia component 1 [file mmc1.docx]

**ADDITIONAL FILE 1**

Comparative structure activity and target exploration of 1,2-diphenylethynes in *Haemonchus contortus* and *Caenorhabditis elegans*

Harrison T. Shanley^a,b^, Aya C. Taki^a^, Nghi Nguyen^b^, Tao Wang^a^, Joseph J. Byrne^a^, Ching-Seng Ang^c^*,* Michael G. Leeming^c^, Nicholas Williamson^c^, Bill C.H. Chang^a^, Abdul Jabbar^a^, Brad E. Sleebs^a,b*^, Robin B. Gasser^a*^

^a^ *Department of Veterinary Biosciences, Melbourne Veterinary School, Faculty of Science, The University of Melbourne, Victoria, Australia*

^b^ *Walter and Eliza Hall Institute of Medical Research, Victoria, Australia*

^c^ *Melbourne Mass Spectrometry and Proteomics Facility, The Bio21 Molecular Science and Biotechnology Institute, The University of Melbourne, Victoria, Australia*

* Corresponding authors. Department of Veterinary Biosciences, Melbourne Veterinary School, Faculty of Science, The University of Melbourne, Parkville, Victoria 3010, Australia; Walter and Eliza Hall Institute of Medical Research, Parkville, Victoria 3052, Australia.

*E-mail addresses:* [sleebs@wehi.edu.au](mailto:sleebs@wehi.edu.au) (B.E. Sleebs); [robinbg@unimelb.edu.au](mailto:robinbg@unimelb.edu.au) (R.B. Gasser).

**Index**

Page

2 Figure S1 *H. contortus* larvae dose-response curves.

3 Figure S2 *C. elegans* larvae dose-response curves.

4 Figure S3 Cytotoxicity dose-response curves.

5 Figure S4 Mitotoxicity dose-response curves.

6 Table S1 TPP statistics summary (*H. contortus*).

6 Table S2 TPP statistics summary (*C. elegans*).

7 S5 Chemistry procedures and compound characterisation.

15 Figure S6 The ^1^H,^13^C and ^19^F spectra, and HPLC traces, of UMW-9729 and all analogues.

**Supplementary Figure 1.** The potencies of UMW-9729 and six active derivative compounds (**12**, **14**, **15**, **16**, **18** and **25**) against exsheathed third-stage larvae of *Haemonchus contortus* with reference to two control compounds (monepantel and moxidectin). The dose-response curve shows the reduction of *H. contortus* motility at 90 h. Data points represent three independent experiments conducted in triplicate; the mean ± the standard error of the mean (SEM).

******

**Supplementary Figure 2.** The potencies of UMW-9729 and six active derivative compounds (**12**, **14**, **15**, **16**, **18** and **25**) against young adults of *Caenorhabditis elegans* with reference to two control compounds (monepantel and moxidectin). The dose-response curve shows the reduction of *C. elegans* motility at 40 h. Data points represent three independent experiments conducted in triplicate; the mean ± the standard error of the mean (SEM).


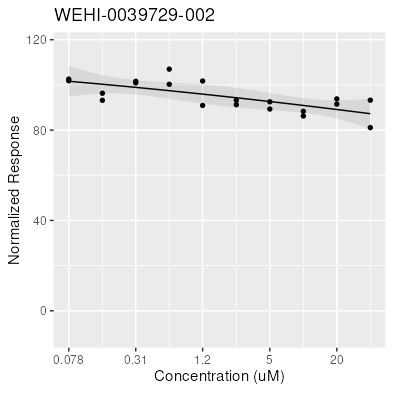


**14**

**12**

**UMW-9729**


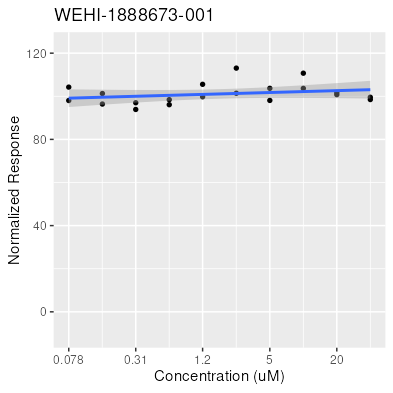

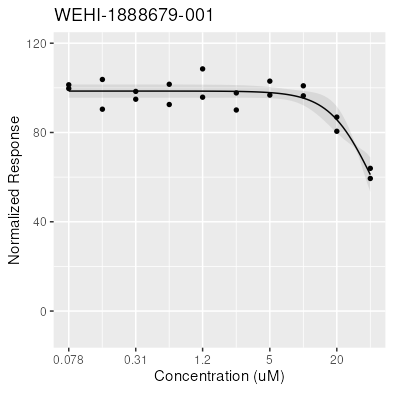


**18**

**16**

**15**


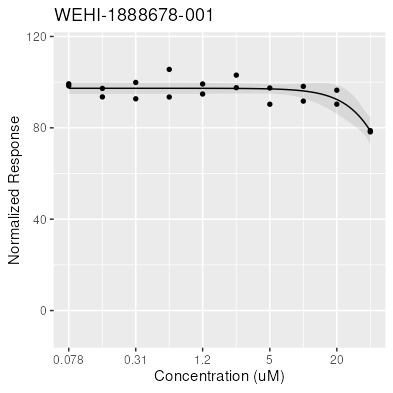

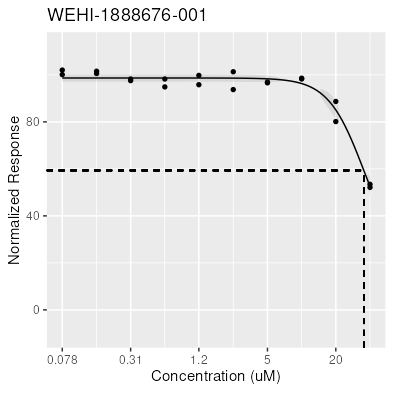

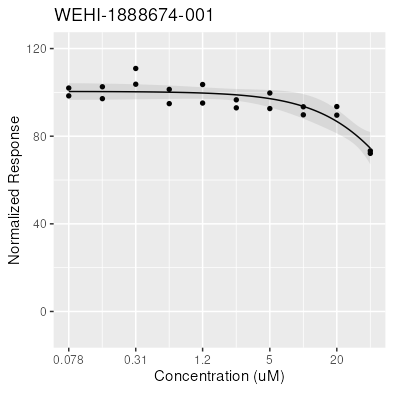

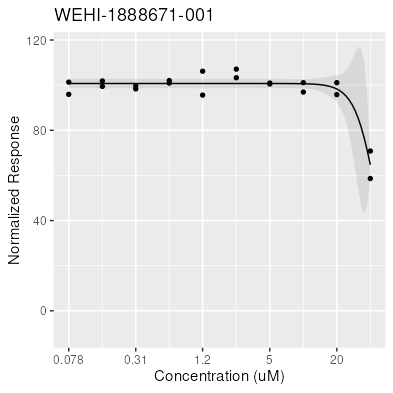


**25**

**Supplementary Figure 3.** Cytotoxicity assessment of UMW-9729 and six active derivative compounds (**12**, **14**, **15**, **16**, **18** and **25**) on human hepatoma (HepG2) cells. For each compound, the half-maximal cytotoxic concentration (CC_50_) was established *via* a cell-viability assay (using CellTiter-Glo^®^) after 48 h of incubation. Data was corrected with reference to a positive control (bortezomib, 10 μM) and a negative control (0.5% dimethylsulfoxide, DMSO). Each data point is presented in technical replicates.

**Supplementary Figure 4.** Mitotoxicity assessment of UMW-9729 and six active derivative compounds (**12**, **14**, **15**, **16**, **18** and **25**), and two reference compounds (monepantel and moxidectin) on human hepatoma (HepG2) cells. For each compound, the half-maximal mitotoxic concentration (MC_50_) was established *via* a cell-viability assay after 48 h of incubation. Crystal violet staining was used to measure the absorbance (595 nm) of treated cells, which was negative- (blank) and baseline- (100% cell viability) corrected. Data points represent triplicates and are presented as mean ± the standard deviation (SD).

**Supplementary Table 1.** The p-values, adjusted p-values and F-statistics of *Haemonchus contortus* proteins identified in a thermal proteome profiling assay with UMW-9729.

**Supplementary Table 2.** The p-values, adjusted p-values and F-statistics of *Caenorhabditis elegans* proteins identified in a thermal proteome profiling assay with UMW-9729.

| **Protein** | **p-Value** | **p-Value (Benjamini-Hochberg-adjusted)** | **F-statistic** |
| --- | --- | --- | --- |
| HCON_00134350 | 1.99 × 10^-7^ | 3.30 × 10^-4^ | 39.7 |
| HCON_00021470 | 3.07 × 10^-7^ | 3.34 × 10^-4^ | 37.3 |
| HCON_00099760 | 3.86 × 10^-6^ | 2.52 × 10^-3^ | 25.1 |

| **Protein** | **p-Value** | **p-Value (Benjamini-Hochberg-adjusted)** | **F-statistic** |
| --- | --- | --- | --- |
| F30A10.9 | 3.40 × 10^-8^ | 7.99 × 10^-5^ | 64.2 |
| F15B9.8 | 8.92 × 10^-8^ | 1.27 × 10^-4^ | 55.6 |
| PINN-4 | 5.76 × 10^-7^ | 5.41 × 10^-4^ | 41.9 |
| UBL-5 | 1.88 × 10^-6^ | 1.48 × 10^-3^ | 34.8 |
| D1086.10 | 6.39 × 10^-6^ | 3.33 × 10^-3^ | 28.5 |
| PGP-1 | 8.25 × 10^-6^ | 3.53 × 10^-3^ | 27.4 |
| H14N18.4 | 8.25 × 10^-6^ | 3.53 × 10^-3^ | 27.4 |
| B0361.6 | 1.71 × 10^-5^ | 5.87 × 10^-3^ | 24.2 |
| DNC-4 | 1.85 × 10^-5^ | 5.87 × 10^-3^ | 23.9 |
| UNC-11 | 2.27 × 10^-5^ | 6.28 × 10^-3^ | 23.0 |
| GST-15 | 2.64 × 10^-5^ | 6.30 × 10^-3^ | 22.4 |
| ZNF-598 | 2.68 × 10^-5^ | 6.30 × 10^-3^ | 22.4 |
| C01G6.4 | 3.63 × 10^-5^ | 7.10 × 10^-3^ | 21.2 |
| TRXR-1 | 4.75 × 10^-5^ | 8.93 × 10^-3^ | 20.2 |

**Supplementary 5.** Chemistry procedures and compound characterisation

**General procedure A**: Compound **2** (0.5 mmol) was dissolved in DMF (2 mL) and degassed with N_2_. An ethynylbenzene derivative (0.75 mmol), CuI (0.025 mmol) and DIPEA (1.5 mmol) were then added to the mixture, which was then degassed with N_2_. Subsequently, Pd(dppf)Cl_2_ (0.005 mmol) was added, and the solution was degassed with N_2_ and allowed to stir at 45 ^°^C for 24 h. The reaction was quenched with 1M NH_4_Cl (2.5 mL) and diluted with EtOAc (10 mL). Brine (5 mL) was added, the solution extracted with EtOAc (3 × 4 mL) and the combined organic layers dried over anhydrous Na_2_SO_4_, filtered, and concentrated *in vacuo* to the give the crude product. The product was then purified using silica gel chromatography gradient eluting with 0–40 % DCM/*n*-heptane.

*5-(4-(Phenylethynyl)phenyl)-1H-pyrazole (****1****).* General procedure A was followed using ethynylbenzene (22 mg), giving compound **1** as a yellow solid in 71 % yield. **^1^H NMR** (300 MHz, DMSO-*d_6_*) δ 13.43 (s, 0.2H, NH tautomer), 13.02 (s, 0.8H, NH tautomer), 7.89 (d, *J* 8.0 Hz, 2H), 7.82 (s, 1H), 7.57 (m, 4H), 7.50–7.36 (m, 3H), 6.79 (d, *J* 2.2 Hz, 1H). **^13^C NMR** (75 MHz, DMSO-*d_6_*) δ 149.31, 134.12, 131.74, 131.34, 130.09, 128.77 (two overlapping **C**-H peaks, determined *via* ^1^H–^13^C HSQC, **Additional File 1: Fig. S7**), 125.29, 122.37, 120.81, 102.21, 89.71, 89.53. **HPLC** Rt (min) 2.462. **LCMS** (*m/z*) 245.2 (M + H^+^). **HRMS** **acquired**: (M + H^+^) 245.1074; C_17_H_12_N_2_ requires (M + H^+^), 245.1073.

*5-(4-Iodophenyl)-1H-pyrazole (****2****).* A solution of 1-(4-iodophenyl)ethanone (5 g, 20 mmol) and *N,N*-dimethylformamide dimethyl acetal in DMF (13 mL, 101 mmol) was stirred at 80 ^°^C for 12 h. The reaction was then cooled to room temperature, diluted with EtOAc (25 mL) and washed with water (15 mL) and citric acid (10 % *w*/*v*, 10 mL). The organic layer was then collected and concentrated *in vacuo*, prior to dissolution in EtOH (10 mL) and treatment with hydrazine monohydrate (3 mL, 61 mmol). The reaction was then stirred at 70 ^°^C for 12 h, cooled to room temperature and subsequently poured into ice-water (50 mL). The precipitate was then collected, washed with ice-water (4 × 5 mL) and dried to provide compound **2** as a yellow solid in 78 % yield. **^1^H NMR** (300 MHz, DMSO-*d_6_*) δ 13.35 (s, 0.2H, NH tautomer), 12.96 (s, 0.8H, NH tautomer), 7.75 (d, *J* 8.9 Hz, 3H), 7.62 (d, *J* 8.0 Hz, 2H), 6.72 (d, *J* 2.2 Hz, 1H). **HPLC** Rt (min) 2.151. **LCMS** (*m/z*) 271.0 (M + H^+^).

*2-Bromo-5-(1H-pyrazol-5-yl)pyridine (****3****).* A solution of 1-(6-bromopyridin-3-yl)ethanone (200 mg, 1 mmol) and *N,N*-dimethylformamide dimethyl acetal in DMF (0.66 mL, 5 mmol) was stirred at 80 ^°^C for 12 h. The reaction was then cooled to room temperature, dissolved in EtOH (2 mL) and treated with hydrazine monohydrate (0.15 mL, 3 mmol). The reaction was then stirred at 70 ^°^C for 12 h, cooled to room temperature, concentrated *in vacuo*, then redissolved in EtOAc (3 × 5 mL) and washed with water (5 mL). The crude mixture was then purified *via* silica gel chromatography (0–20 % EtOAc/n-heptane) to give compound **3** as a brown solid in 51 % yield. **^1^H NMR** (300 MHz, DMSO-*d_6_*) δ 13.52 (s, 0.2 H, NH tautomer), 13.14 (s, 0.8H, NH tautomer), 8.83 (d, *J* 2.5 Hz, 1H), 8.13 (dd, *J* 8.3, 2.5 Hz, 1H), 7.85 (t, *J* 1.9 Hz, 1H), 7.67 (d, *J* 8.3 Hz, 1H), 6.87 (dd, *J* 2.5, 1.1 Hz, 1H). **HPLC** Rt (min) 1.544. **LCMS** (*m/z*) 224.2 (M + H^+^), 226.2 (M + H^+^).

*5-(3-Bromophenyl)-1H-pyrazole (****4****)*. A solution of 1-(3-bromophenyl)ethanone (240 mg, 1.2 mmol) and *N,N*-dimethylformamide dimethyl acetal in DMF (0.8 mL, 6 mmol) was stirred at 80 ^°^C for 12 h. The reaction mixture was allowed too cool to room temperature and diluted in EtOH (2 ml). Subsequently, hydrazine monohydrate (0.8 mL, 3.6 mmol) was added, and the reaction mixture was stirred at 70 ^°^C for 12 h. The solution was allowed to cool to room temperature and concentrated *in vacuo*. The concentrate was dissolved in EtOAc (5 mL) and water (5 mL), extracted with EtOAc (3 × 5 mL), with the organic layers combined, dried over anhydrous Na_2_SO_4_, filtered, and concentrated *in vacuo* to the give the crude product. The product was then purified using silica gel chromatography gradient eluting with 0–20 % EtOAc/*n*-heptane, giving **4** as an orange solid in 68 % yield. **^1^H NMR** (300 MHz, DMSO-*d_6_*) δ 13.39 (s, 0.2H, NH tautomer), 13.02 (s, 0.8H, NH tautomer), 8.01 (t, *J* 1.8 Hz, 1H), 7.82 (d, *J* 8.4 Hz, 2H), 7.47 (d, *J* 8.0 Hz, 1H), 7.35 (t, *J* 7.8 Hz, 1H), 6.80 (d, *J* 2.3 Hz, 1H). **HPLC** Rt (min) 2.051. **LCMS** (*m/z*) 225.2 (M + H^+^).

*5-(4-Bromophenyl)-1-methyl-1H-pyrazole (****5****).* 1-Methyl-1*H*-pyrazole-5-boronic acid pinacol ester (123 mg, 0.59 mmol), 1-bromo-4-iodobenzene (186 mg, 0.66 mmol) and K_2_CO_3_ (273 mg, 2.0 mmol) were dissolved in a solvent system of 1,4-dioxane (9 mL)/water (1 mL). The solution was degassed with N_2_ prior to addition of Pd(dppf)Cl_2_.DCM (27 mg, 0.03 mmol). The solution was again degassed under N_2_ and allowed to stir at 90 ^°^C overnight. The mixture was then filtered through Celite^®^, extracted with EtOAc (10 mL) and concentrated *in vacuo* to give the crude. The product was then purified using silica gel chromatography gradient eluting with 0–20 % EtOAc/*n*-heptane, giving compound **5** as a white in 66 % yield. **^1^H NMR** (300 MHz, CDCl_3_) δ 7.62–7.56 (m, 2H), 7.51 (d, *J* 1.9 Hz, 1H), 7.32–7.26 (m, 2H), 6.30 (d, *J* 1.9 Hz, 1H), 3.87 (s, 3H). **HPLC** Rt (min) 2.222. **LCMS** (*m/z*) 237.2, 239.2 (M + H^+^).

*5-(4-(2-(2-Methoxyphenyl)ethynyl)phenyl)-1H-pyrazole (****6****).* General procedure A was followed using 1-ethynyl-2-methoxybenzene (98 mg), giving compound **6** as an orange solid in 66 % yield. **^1^H NMR** (300 MHz, DMSO-*d_6_*) δ 13.40 (s, 0.2H, NH tautomer), 13.01 (s, 0.8H, NH tautomer), 7.86 (d, *J* 7.9 Hz, 2H), 7.81 (s, 1H), 7.55 (d, *J* 8.0 Hz, 2H), 7.49 (dd, *J* 7.6, 1.7 Hz, 1H), 7.39 (ddd, *J* 8.9, 7.5, 1.8 Hz, 1H), 7.09 (d, *J* 8.3 Hz, 1H), 6.98 (td, *J* 7.5, 1.0 Hz, 1H), 6.78 (d, *J* 2.3 Hz, 1H), 3.86 (s, 3H). **^13^C NMR** (75 MHz, DMSO-*d_6_*) δ 159.66, 133.09, 131.65, 130.40, 125.32, 121.49, 120.55, 111.40, 111.36, 102.35, 93.06, 86.69, 55.72. ^1^H–^13^C HSQC experiment indicates pyrazole **C**-H is obscured (**Additional File 1: Fig. S7**) two quaternary carbons remain unresolved. **HPLC** Rt (min) 2.455. **LCMS** (*m/z*) 275.2 (M + H^+^). **HRMS** **acquired**: (M + H^+^) 275.1180; C_18_H_14_N_2_O requires (M + H^+^), 275.1179.

*5-(4-((2-(Trifluoromethyl)phenyl)ethynyl)phenyl)-1H-pyrazole (****7****).* General procedure A was followed using 1-ethynyl-2-(trifluoromethyl)benzene (0.1 mL), giving compound **7** as a white solid in 63 % yield. **^1^H NMR** (300 MHz, DMSO-*d_6_*) δ 13.01 (s, 1H), 7.95–7.87 (m, 2H), 7.87–7.80 (m, 2H), 7.79–7.68 (m, 2H), 7.66–7.61 (m, 1H), 7.61–7.54 (m, 2H), 6.80 (d, *J* 2.2 Hz, 1H). **^13^C NMR** (75 MHz, DMSO-*d_6_*) δ 147.72, 133.82, 132.55, 132.34, 132.20, 131.82, 129.83 (q, *J_CF_* 29.8 Hz), 128.96, 126.08 (q, *J_CF_* 5.1 Hz), 125.44, 123.05 (q, *J_CF_* 273.3 Hz), 120.35, 120.29 (q, *J_CF_* 2.5 Hz), 102.66, 94.82, 85.62. **^19^F NMR** (282 MHz, DMSO-*d_6_*) δ -60.69. **HPLC** Rt (min) 2.719. **LCMS** (*m/z*) 313.2 (M + H^+^). **HRMS acquired**: (M + H^+^) 313.0938; C_18_H_11_F_3_N_2_ requires (M + H^+^), 313.0947.

*5-(4-((2-(Fluorophenyl)ethynyl)phenyl)-1H-pyrazole (****8****).* General procedure A was followed using 1-ethynyl-2-fluorobenzene (0.02 mL), giving compound **8** as an off-white solid in 84 % yield. **^1^H NMR** (300 MHz, DMSO-*d_6_*) δ 13.43 (s, 0.2H, NH tautomer), 13.02 (s, 0.8H, NH tautomer), 7.90 (d, *J* 8.0 Hz, 2H), 7.82 (s, 1H), 7.67–7.54 (m, 3H), 7.53–7.41 (m, 1H), 7.34 (ddd, *J* 9.7, 8.4, 1.1 Hz, 1H), 7.27 (td, *J* 7.5, 1.2 Hz, 1H), 6.80 (d, *J* 2.2 Hz, 1H).**^13^C NMR** (75 MHz, DMSO-*d_6_*) δ 161.79 (d, *J_F_* 249.4 Hz), 149.25, 134.49, 133.42, 131.81, 131.01 (d, *J_F_* 7.8 Hz), 130.12, 125.35, 124.84 (d, *J_F_* 3.5 Hz), 120.33, 115.75 (d, *J_F_* 20.5 Hz), 110.72 (d, *J_F_* 15.9 Hz), 102.27, 94.45, 82.89*.* **HPLC** Rt (min) 2.684. **^19^F NMR** (282 MHz, DMSO-*d_6_*) δ -110.11. **LCMS** (*m/z*) 279.2 (M + H^+^). **HRMS acquired**: 263.0980 (M + H^+^); C_17_H_11_FN_2_ requires (M + H^+^), 263.0979.

*5-(4-((2-Chlorophenyl)ethynyl)phenyl)-1H-pyrazole (****9****).* General procedure A was followed using 1-chloro-2-ethynylbenzene (100 mg), giving compound **9** as an off-white solid in 70 % yield. **^1^H NMR** (300 MHz, DMSO- *d_6_*) δ 13.04 (s, 1H), 7.96–7.82 (m, 2H), 7.77 (s, 1H), 7.70–7.65 (m, 1H), 7.65–7.54 (m, 3H), 7.47–7.34 (m, 2H), 6.79 (d, *J* 2.3 Hz, 1H)*.* **^13^C NMR** (75 MHz, DMSO-*d_6_*) δ 134.59, 133.31, 131.87, 130.27, 129.38, 127.36, 125.36, 122.08, 120.50, 102.45, 94.49, 86.45*.* ^1^H–^13^C HSQC experiment indicates pyrazole **C**-H is obscured (**Additional File 1: Fig. S7**), two quaternary carbons remain unresolved. **HPLC** Rt (min) 2.688. **LCMS** (*m/z*) 279.2 (M + H^+^). **HRMS acquired**: (M + H^+^) 279.0686; C_17_H_11_ClN_2_ requires (M + H^+^), 279.0684.

*2-((4-(1H-Pyrazol-5-yl)phenyl)ethynyl)pyridine (****10****).* General procedure A was followed using 2-ethynlpyridine (76 mg), giving compound **10** as a yellow solid in 39 % yield. **^1^H NMR** (300 MHz, DMSO- *d_6_*) δ 13.03 (s, 1H), 8.62 (ddd, *J* 4.9, 1.8, 1.0 Hz, 1H), 7.94–7.87 (m, 2H), 7.85 (dd, *J* 7.7, 1.8 Hz, 1H), 7.79 (s, 1H), 7.65 (dd, *J* 8.2, 2.0 Hz, 3H), 7.42 (ddd, *J* 7.7, 4.9, 1.2 Hz, 1H), 6.81 (d, *J* 2.2 Hz, 1H). **^13^C NMR** (75 MHz, DMSO-*d_6_*) δ 150.17, 142.33, 136.81, 132.13, 127.29, 125.37, 123.49, 120.02, 102.50, 89.46, 88.51. ^1^H–^13^C HSQC experiment indicates pyrazole **C**-H is obscured (**Additional File 1: Fig. S7**), two quaternary carbons remain unresolved. **HPLC** Rt (min) 1.847. **LCMS** (*m/z*) 246.2 (M + H^+^). **HRMS acquired**: (M + H^+^) 246.1027; C_16_H_11_N_3_ requires (M + H^+^), 246.1026.

*5-(4-(m-Tolylethynyl)phenyl)-1H-pyrazole (****11****).* General procedure A was followed using 1-ethynyl-3-methylbenzene (0.04 mL), giving compound **11** as a white solid in 42 % yield. **^1^H NMR** (300 MHz, DMSO- *d_6_*) δ 13.40 (s, 0.2H, NH tautomer), 12.99 (s, 0.8H, NH tautomer), 7.87 (d, *J* 8.0 Hz, 2H), 7.81 (s, 1H), 7.57 (d, *J* 8.0 Hz, 2H), 7.41–7.28 (m, 3H), 7.23 (d, *J* 7.1 Hz, 1H), 6.78 (d, *J* 2.2 Hz, 1H), 2.33 (s, 3H). **^13^C NMR** (75 MHz, DMSO-*d_6_*) δ 149.30, 138.10, 134.04, 131.76, 131.70, 130.07, 129.52, 128.65, 128.41, 125.29, 122.20, 120.90, 102.19, 89.88, 89.21, 20.71. **HPLC** Rt (min) 2.612. **LCMS** (*m/z*) 259.2 (M + H^+^). **HRMS acquired**: (M + H^+^) 259.1229; C_18_H_14_N_2_ requires (M + H+), 259.1230.

*5-(4-((3-(Trifluoromethyl)phenyl)ethynyl)phenyl)-1H-pyrazole (****12****).* General procedure A was followed using 1-ethynyl-3-(trifluoromethyl)benzene (0.03 mL), giving compound **12** as a white solid in 44 % yield. **^1^H NMR** (300 MHz, DMSO-*d_6_*) δ 13.41 (s, 0.2H, NH tautomer), 13.02 (s, 0.8H, NH tautomer), 7.95–7.83 (m, 4H), 7.82–7.73 (m, 3H), 7.72–7.60 (m, 3H), 6.80 (d, *J* 2.2 Hz, 1H)*.* **^13^C NMR** (75 MHz, DMSO-*d_6_*) δ 149.23, 135.16, 134.53, 131.99, 130.15, 130.01, 129.70 (q, *J_CF_* 32.0 Hz), 127.79 (q, *J_CF_* 3.8 Hz), 125.33, 125.21 (q, *J_CF_* 3.6 Hz), 123.74 (q, *J_CF_* 272.5 Hz), 123.54, 120.26, 102.34, 91.12, 88.13. **^19^F NMR** (282 MHz, DMSO-*d_6_*) δ -61.10. **HPLC** Rt (min) 2.822. **LCMS** (*m/z*) 313.2 (M + H^+^). **HRMS acquired**: (M + H^+^) 313.0939; C_18_H_11_F_3_N_2_ requires (M + H^+^), 313.0947.

*5-(4-((3-(Trifluoromethoxy)phenyl)ethynyl)phenyl)-1H-pyrazole (****13****).* General procedure A was followed using 1-ethynyl-3-(trifluoromethoxy)benzene (0.03 mL), giving compound **13** as a white solid in 58 % yield. **^1^H NMR** (300 MHz, DMSO-*d_6_*) δ 13.43 (s, 0.2H, NH tautomer), 13.02 (s, 0.8H, NH tautomer), 7.89 (d, *J* 7.9 Hz, 2H), 7.81 (s, 1H), 7.66–7.50 (m, 5H), 7.47–7.38 (m, 1H), 6.79 (d, *J* 2.2 Hz, 1H). **^13^C NMR** (75 MHz, DMSO-*d_6_*) δ 149.26, 148.36 (q, *J_CF_* 1.7 Hz), 134.52, 131.95, 130.91, 130.54, 130.12, 125.32, 124.46, 123.62, 121.54, 120.24, 120.03 (q, *J_CF_* 257.0 Hz), 102.30, 90.98, 88.02. **^19^F NMR** (282 MHz, DMSO-*d_6_*) δ -56.52. **HPLC** Rt (min) 2.749. **LCMS** (*m/z*) 329.2 (M + H^+^). **HRMS acquired**: 329.0897 (M + H^+^); C_18_H_11_F_3_N_2_O requires (M + H^+^), 329.0896.

*5-(4-((3-(Fluorophenyl)ethynyl)phenyl)-1H-pyrazole (****14****).* General procedure A was followed using 1-ethynyl-3-fluorobenzene (0.04 mL), giving compound **14** as a yellow solid in 72 % yield. **^1^H NMR** (300 MHz, DMSO-*d_6_*) δ 13.03 (s, 1H), 7.88 (d, *J* 8.1 Hz, 2H), 7.77 (s, 1H), 7.60 (d, *J* 8.1 Hz, 2H), 7.52–7.38 (m, 3H), 7.27 (dddd, *J* 9.3, 8.0, 2.7, 1.3 Hz, 1H), 6.79 (d, *J* 2.2 Hz, 1H). **^13^C NMR** (75 MHz, DMSO-*d_6_*) δ161.90 (d, *J_F_* 244.7 Hz), 131.90, 130.86 (d, *J_F_* 8.9 Hz), 127.71 (d, *J_F_* 2.9 Hz), 125.32, 124.34 (d, *J_F_* 9.7 Hz), 120.48, 117.88 (d, *J_F_* 22.9 Hz), 116.02 (d, *J_F_* 21.1 Hz), 102.41, 90.42, 88.49. ^1^H–^13^C HSQC experiment indicates pyrazole **C**-H is obscured (**Additional File 1: Fig. S7**), two quaternary carbons remain unresolved. **^19^F NMR** (282 MHz, DMSO-*d_6_*) δ -112.11. **HPLC** Rt (min) 2.516. **LCMS** (*m/z*) 263.2 (M + H^+^). **HRMS acquired**: 263.0972 (M + H^+^); C_17_H_11_FN_2_ requires (M + H^+^), 263.0979 .

*5-(4-((3-Chlorophenyl)ethynyl)phenyl)-1H-pyrazole (****15****).* General procedure A was followed using 1-chloro-3-ethynyl-benzene (0.025 mL), giving compound **15** as a white solid in 50 % yield.**^1^H NMR** (300 MHz, DMSO- *d_6_*) δ 13.04 (s, 1H), 7.88 (d, *J* 8.0 Hz, 2H), 7.77 (s, 1H), 7.65–7.56 (m, 3H), 7.55–7.39 (m, 3H), 6.79 (d, *J* 2.2 Hz, 1H).**^13^C NMR** (75 MHz, DMSO-*d_6_*) δ 133.36, 131.94, 131.55, 131.42, 130.76, 130.61, 130.00, 128.82, 128.67, 125.33, 124.36, 120.46, 102.43, 90.79, 88.28. **HPLC** Rt (min) 2.455. **LCMS** (*m/z*) 263.2 (M + H^+^). **HRMS acquired**: 279.0688 (M + H^+^); C_17_H_11_ClN_2_ requires (M + H^+^), 279.0684.

*3-((4-(1H-Pyrazol-5-yl)phenyl)ethynyl)pyridine (****16****).* General procedure A was followed using 3-ethynylpyridine (21 mg), giving compound **16** as a pale yellow solid in 73 % yield. **^1^H NMR** (300 MHz, DMSO-*d_6_*) δ 13.42 (s, 0.2H, NH tautomer), 13.03 (s, 0.8H, NH tautomer), 8.77 (dd, *J* 2.2, 0.9 Hz, 1H), 8.59 (dd, *J* 4.9, 1.6 Hz, 1H), 7.98 (dt, *J* 7.9, 1.9 Hz, 1H), 7.89 (d, *J* 8.0 Hz, 2H), 7.81 (s, 1H), 7.62 (d, *J* 7.9 Hz, 2H), 7.46 (ddd, *J* 7.9, 4.9, 0.9 Hz, 1H), 6.79 (d, *J* 2.2 Hz, 1H). **^13^C NMR** (75 MHz, DMSO-*d_6_*) δ 151.58, 149.32, 148.94, 138.48, 134.53, 131.92, 130.11, 125.33, 123.62, 120.26, 119.48, 102.33, 92.48, 86.57. **HPLC Rt** (min) 1.843. **LCMS** (*m/z*) 246.2 (M + H^+^). **HRMS acquired**: (M + H^+^) 246.1020; C_16_H_11_N_3_ requires (M + H^+^), 246.1026.

*3-((4-(1H-Pyrazol-5-yl)phenyl)ethynyl)phenol (****17****).* General procedure A was followed using 3-ethynylphenol (87 mg), giving compound **17** a white solid in 21 % yield. **^1^H NMR** (300 MHz, DMSO-*d_6_*) δ 13.42 (s, 0.2H, NH tautomer), 13.02 (s, 0.8H, NH tautomer), 9.74 (s, 1H), 7.87 (d, *J* 8.0 Hz, 2H), 7.81 (s, 1H), 7.58 (d, *J* 8.0 Hz, 2H), 7.22 (t, *J* 7.9 Hz, 1H), 6.99 (dt, *J* 7.5, 1.2 Hz, 1H), 6.94 (t, *J* 2.0 Hz, 1H), 6.84 (dd, *J* 8.1, 2.5 Hz, 1H), 6.78 (d, *J* 2.2 Hz, 1H). **^13^C NMR** (75 MHz, DMSO-*d_6_*) δ 157.43, 149.35, 134.06, 131.82, 130.13, 129.94, 125.31, 123.26, 122.27, 120.97, 117.77, 116.34, 102.28, 89.96, 88.94. **HPLC** Rt (min) 2.178. **LCMS** (*m/z*) 261.2 (M + H^+^). **HRMS** **acquired**: (M + H^+^) 261.1022; C_17_H_12_N_2_O requires (M + H^+^), 261.1022.

*5-(4-(p-Tolylethynyl)phenyl)-1H-pyrazole (****18****).* General procedure A was followed using 1-ethynyl-4-methylbenzene (0.04 mL), giving compound **18** as a white solid in 63 % yield. **^1^H NMR** (300 MHz, DMSO-*d_6_*) δ 13.40 (s, 0.2H, NH tautomer), 13.00 (s, 0.8H, NH tautomer), 7.86 (d, *J* 8.0 Hz, 2H), 7.81 (s, 1H), 7.56 (d, *J* 7.9 Hz, 2H), 7.45 (d, 2H), 7.23 (d, *J* 7.9 Hz, 2H), 6.78 (d, *J* 2.2 Hz, 1H), 2.33 (s, 3H). **^13^C NMR** (75 MHz, DMSO-*d_6_*) δ 149.37, 138.53, 133.95, 131.67, 131.25, 130.07, 129.39, 125.28, 121.07, 119.34, 102.19, 89.94, 88.90, 21.04. **HPLC** Rt (min) 2.606. **LCMS** (*m/z*) 259.2 (M + H^+^). **HRMS acquired**: 259.1233 (M + H^+^); C_18_H_14_N_2_ requires (M + H^+^), 259.1230.

*5-(4-((4-Methoxyphenyl)ethynyl)phenyl)-1H-pyrazole (****19****).* General procedure A was followed using 1-ethynyl-4-methoxybenzene (98 mg), giving compound **19** as an orange solid in 40 % yield. **^1^H NMR** (300 MHz, DMSO-*d_6_*) δ 13.32 (s, 0.2H, NH tautomer), 13.00 (s, 0.8H, NH tautomer), 7.85 (d, *J* 8.0 Hz, 2H), 7.78 (s, 1H), 7.55 (d, *J* 8.2 Hz, 2H), 7.50 (d, *J* 8.7 Hz, 2H), 7.04–6.93 (m, 2H), 6.78 (d, *J* 2.2 Hz, 1H), 3.79 (s, 3H). **^13^C NMR** (75 MHz, DMSO-*d_6_*) δ 159.55, 132.93, 131.56, 125.26, 121.34, 114.43, 114.27, 102.29, 89.96, 88.11, 55.29. Pyrazole **C**-H and two quaternary carbons remain unresolved. **HPLC** Rt (min) 2.565. **LCMS** (*m/z*) 275.2 (M + H^+^). **HRMS acquired**: (M + H^+^) 275.1180; C_18_H_14_N_2_O requires (M + H^+^), 275.1179.

*5-(4-((4-Chlorophenyl)ethynyl)phenyl)-1H-pyrazole (****20****).* General procedure A was followed using 1-chloro-4-ethynylbenzene (0.1 mL), giving compound **20** as a yellow solid in 71 % yield. **^1^H NMR** (300 MHz, DMSO- *d_6_*) δ) δ 13.43 (s, 0.2H, NH tautomer), 13.02 (s, 0.8H, NH tautomer), 7.88 (d, *J* 8.0 Hz, 2H), 7.82 (s, 1H), 7.62–7.54 (m, 4H), 7.54–7.44 (m, 2H), 6.79 (d, *J* 2.2 Hz, 1H). **^13^C NMR** (75 MHz, DMSO-*d_6_*) δ 149.26, 134.31, 133.45, 133.05, 131.81, 130.09, 128.93, 125.30, 121.25, 120.51, 102.25, 90.57, 88.56. **HPLC** Rt (min) 2.812. **LCMS** (*m/z*) 279.2 (M + H^+^). **HRMS** **acquired**: (M + H^+^), 279.0685; C_17_H_11_ClN_2_ requires (M + H^+^), 279.0684.

*4-((4-(1H-Pyrazol-5-yl)phenyl)ethynyl)benzonitrile (****21****).* General procedure A was followed using 4-ethynylbenzonitrile (0.04 mL), giving compound **21** as a yellow solid in 54 % yield. **^1^H NMR** (300 MHz, DMSO-*d_6_*) δ (300 MHz, DMSO) δ 13.02 (s, 1H), 7.89 (d, *J* 8.2 Hz, 4H), 7.77 (s, 1H), 7.74 (d, *J* 8.3 Hz, 2H), 7.63 (d, *J* 8.3 Hz, 2H), 6.81 (d, *J* 2.1 Hz, 1H).**^13^C NMR** (75 MHz, DMSO-*d_6_*) δ 132.60, 132.09 (two overlapping **C**-H peaks, determined *via* ^1^H–^13^C HSQC, **Additional File 1: Fig. S7**), 127.26, 125.35, 120.07, 118.46, 110.88, 102.57, 93.54, 88.44. ^1^H–^13^C HSQC experiment indicates pyrazole **C**-H is obscured (**Additional File 1: Fig. S7**), two quaternary carbons remain unresolved. **HPLC** Rt (min) 2.372. **LCMS** (*m/z*) 270.2 (M + H^+^). **HRMS acquired**: (M + H^+^) 270.1019; C_18_H_11_N_3_ requires (M + H^+^), 270.1026.

*4-((4-(1H-Pyrazol-5-yl)phenyl)ethynyl)pyridine (****22****).* General procedure A was followed using 4-ethynylpyridine hydrochloride (103 mg), giving compound **22** as a white solid in 27 % yield. **^1^H NMR** (300 MHz, DMSO-*d_6_*) δ 13.43 (s, 0.2H, NH tautomer), 13.02 (s, 0.8H, NH tautomer), 8.67–8.59 (m, 2H), 7.91 (d, *J* 7.9 Hz, 2H), 7.82 (s, 1H), 7.64 (d, *J* 7.9 Hz, 2H), 7.57–7.49 (m, 2H), 6.81 (d, *J* 2.3 Hz, 1H). **^13^C NMR** (75 MHz, DMSO-*d_6_*) δ 149.97, 149.16, 134.95, 132.17, 130.25, 130.17, 125.37, 125.32, 119.70, 102.39, 93.85, 87.13. **HPLC** Rt (min) 1.479. **LCMS** (*m/z*) 246.2 (M + H^+^). **HRMS acquired**: (M + H^+^) 246.1019; C_16_H_11_N_3_ requires (M + H+), 246.1026.

*5-(4-((Trimethylsilyl)ethynyl)phenyl)-1H-pyrazole (****23****).* General procedure A was followed using ethynyltrimethylsilane (101 mg), giving compound **23** as a white solid in 73 % yield. **^1^H NMR** (300 MHz, DMSO-*d_6_*) δ 12.99 (s, 1H), 7.81 (d, *J* 8.1 Hz, 2H), 7.76 (s, 1H), 7.48 (d, *J* 8.1 Hz, 2H), 6.77 (d, *J* 2.2 Hz, 1H), 0.24 (s, 9H). **HPLC** Rt (min) 2.704. **LCMS** (*m/z*) 241.3 (M + H^+^). **HRMS acquired**: (M + H^+^) 241.1157; C_14_H_16_N_2_Si requires (M + H^+^), 241.1156.

*2-(Phenylethynyl)-5-(1H-pyrazol-5-yl)pyridine (****24****).* 2-Bromo-5-(1*H*-pyrazol-5-yl)pyridine (50 mg, 0.22 mmol, **3**) was dissolved in DMF (2 mL), and the resultant solution was degassed with N_2_. Ethynylbenzene (0.022 mL, 0.22 mmol), CuI (2 mg, 0.01 mmol) and DIPEA (0.11 mL, 0.64 mmol) were subsequently added, and the solution was again degassed with N_2_. Pd(PPh_3_)Cl_2_ (1.6 mg, 0.002 mmol) was then added, the solution degassed with N_2_ and allowed to stir at 45 ^°^C for 24 h. The reaction was quenched with 1M NH_4_Cl (2.5 mL) and diluted with EtOAc (10 mL). Brine (5 mL) was added, the solution extracted with EtOAc (3 × 4 mL) and the combined organic layers dried over anhydrous Na_2_SO_4_, filtered, and concentrated *in vacuo* to the give the crude product. The product was then purified using silica gel chromatography gradient eluting with 0–40 % DCM/*n*-heptane, giving compound **24** as a yellow solid in 49 % yield. **^1^H NMR** (300 MHz, DMSO-*d_6_*) δ 13.57 (s, 0.2H, NH tautomer), 13.17 (s, 0.8H, NH tautomer), 9.08 (s, 1H), 8.22 (dd, *J* 8.2, 2.2 Hz, 1H), 7.87 (s, 1H), 7.68 (d, *J* 8.2 Hz, 1H), 7.61 (ddd, *J* 5.9, 4.6, 2.8 Hz, 2H), 7.51 – 7.42 (m, 3H), 6.91 (d, *J* 2.2 Hz, 1H)*.* **^13^C NMR** (75 MHz, DMSO-*d_6_*) δ 146.89, 140.66, 132.59, 131.67 (two overlapping **C**-H peaks, determined *via* ^1^H–^13^C HSQC, **Additional File 1: Fig. S7**), 130.40, 129.42, 128.90, 127.36, 121.51, 102.69, 89.17, 88.77. One quaternary carbon remains unresolved. **HPLC** Rt (min) 2.045. **LCMS** (*m/z*) 246.2 M + H^+^). **HRMS acquired**: 246.1025 (M + H^+^); C_16_H_11_N_3_ requires (M + H^+^), 246.1026.

*1-Methyl-5-(4-(phenylethynyl)phenyl-1H-pyrazole (****25****).* General procedure A was followed using ethynylbenzene (11 mg), giving compound **25** as a light brown solid in 62 % yield. **^1^H NMR** (300 MHz, DMSO-*d_6_*) δ 7.67 (d, *J* 8.6 Hz, 2H), 7.63–7.55 (m, 4H), 7.49 (d, *J* 1.9 Hz, 1H), 7.47–7.39 (m, 3H), 6.48 (d, *J* 1.9 Hz, 1H), 3.89 (s, 3H). **^13^C NMR** (75 MHz, DMSO-*d_6_*) δ 141.93, 138.06, 131.74, 131.45 (two overlapping **C**-H peaks, determined *via* ^1^H–^13^C HSQC, **Additional File 1: Fig. S7**), 130.31, 129.00, 128.82, 128.62, 122.10, 106.18, 90.41, 88.92, 37.73. **HPLC** Rt (min) 2.670. **LCMS** (*m/z*) 259.2 (M + H^+^). **HRMS acquired**: 259.1229 (M + H^+^); C_18_H_14_N_2_ requires (M + H^+^), 259.1230.

*5-(3-(Phenylethynyl)phenyl-1H-pyrazole (****26****).* 5-(3-Bromophenyl)-1H-pyrazole (20 mg, 0.09 mmol, **4**), ethynylbenzene (0.02 mL, 0.18 mmol), xantphos (4.2 mg, 0.01 mmol) and K_3_PO_4_ (76 mg, 0.36 mmol) were dissolved in toluene (5 mL) and degassed with N_2_. Subsequently, Pd(OAc)_2_ (1 mg, 0.004 mmol) was added and the solution was allowed to stir at 120 ^°^C for 72 h. The reaction mixture was allowed to cool, filtered through Celite^®^, and diluted with EtOAc (5 mL). Brine (5 mL) was added, the solution extracted with EtOAc (3 × 4 mL) and the combined organic layers dried over anhydrous Na_2_SO_4_, filtered, and concentrated *in vacuo* to the give the crude product. The product was then purified using silica gel chromatography gradient eluting with 0–40% DCM/*n*-heptane, giving compound **26** as a clear residue in 34 % yield. **^1^H NMR** (300 MHz, DMSO-*d_6_*) δ 7.99 (q, *J* 1.3 Hz, 1H), 7.86 (ddd, *J* 5.3, 3.4, 1.8 Hz, 1H), 7.75 (d, *J* 2.3 Hz, 1H), 7.62 – 7.55 (m, 2H), 7.53 – 7.39 (m, 5H), 6.81 (d, *J* 2.2 Hz, 1H). N**H** peak not present, possibly due to water in sample. **HPLC** Rt (min) 2.561. **LCMS** (*m/z*) 245.2 (M + H^+^). **HRMS acquired**: 245.1076 (M + H^+^); C_17_H_12_N_2_ requires (M + H^+^), 245.1073.

*5-(4-Ethynylphenyl)-1H-pyrazole (****27****).* 5-(4-((Trimethylsilyl)ethynyl)phenyl)-1H-pyrazole (55 mg, 0.23 mmol, **23**) and K_2_CO_3_ (79 mg, 0.57 mmol) were dissolved in MeOH (2 mL) and allowed to stir at room temperature for 2 h. The mixture was then concentrated *in vacuo* and redissolved in EtOAc (5 mL) and water (5 mL). The crude product was then extracted in EtOAc (3 × 5 mL), the organic layers were combined, dried over anhydrous Na_2_SO_4_ and concentrated *in vacuo* to give the desired product as an orange solid in quantitative yield – no further purification was necessary. **^1^H NMR** (300 MHz, DMSO-*d_6_*) δ 7.87–7.80 (m, 2H), 7.75 (d, *J* 2.2 Hz, 1H), 7.55–7.48 (m, 2H), 6.76 (d, *J* 2.2 Hz, 1H), 4.20 (s, 1H). **^13^C NMR** (75 MHz, DMSO-*d_6_*) δ 147.52, 132.11 (two overlapping **C**-H peaks, determined *via* ^1^H–^13^C HSQC, **Additional File 1: Fig. S7**), 125.23, 120.46, 102.39, 83.56, 81.14. **HPLC** Rt (min) 1.826. **LCMS** (*m/z*) 169.2 (M + H^+^). **HRMS acquired**: (M + H^+^) 169.0757; C_11_H_8_N_2_ requires (M + H^+^), 169.0760.

*5-(4-Phenethylphenyl)-1H-pyrazole (****28****).* A solution of 5-(4-(phenylethynyl)phenyl)-1*H*-pyrazole (20 mg, 0.08 mmol, **5**) in MeOH (2 mL) was degassed with N_2_. Pd/C (1.7 mg, 0.02 mmol) was added, the reaction vial evacuated, and refilled with H_2_. The mixture was then allowed to stir at room temperature for 12 h, after this the H_2_ was evacuated. The solution was subsequently filtered through Celite^®^, washed with MeOH (5 mL) and concentrated *in vacuo*. The product was then purified using silica gel chromatography eluting with 50 % EtOAc/*n*-heptane, giving **28** as a white solid in 69 % yield. **^1^H NMR** (300 MHz, CDCl_3_) δ 8.27 (s, 1H), 7.83–7.73 (m, 2H), 7.69 (d, *J* 2.3 Hz, 1H), 7.43–7.22 (m, 7H), 6.68 (d, *J* 2.2 Hz, 1H), 3.04 (s, 4H). **HPLC** Rt (min) 2.505. **LCMS** (*m/z*) 249.2 (M + H^+^). HRMS acquired: (M + H^+^) 249.1380; C_17_H_16_N_2_ requires (M + H^+^), 249.1386.

*4-Bromo-N’-hydroxybenzimidamide (****29****).* To a solution of 4-bromobenzonitrile (200 mg, 1.1 mmol) in EtOH (10 mL) was added 50% aq. NH_2_OH solution (0.16 mL). The reaction mixture was stirred at 70 ^°^C for 8 h, after which the solution was cooled to room temperature and concentrated *in vacuo* to give compound **29** as white solid in quantitative yield – the product was not purified further. **^1^H NMR** (300 MHz, DMSO-*d_6_*) δ 9.76 (s, 1H), 7.66–7.61 (m, 2H), 7.59–7.51 (m, 2H), 5.87 (s, 2H). **HPLC** Rt (min) 0.648. **LCMS** (*m/z*) 215.2, 217.0 (M + H^+^).

*3-(4-Bromophenyl)-5-methyl-1,2,4-oxadiazole (****30****).* Acetic acid (0.03 mL, 0.47 mmol), HATU (194 mg, 0.51 mmol) and DIPEA (0.2 mL, 1.2 mmol) were dissolved in DMF (1.5 mL) and stirred at room temperature for 30 min. Subsequently, 4-bromo-*N’*-hydroxybenzimidamide (110 mg, 0.51 mmol, **29**) was added and reaction solution was stirred at 100 ^°^C for 12 h. The solution was cooled to room temperature and concentrated *in vacuo* to give the crude product. The product was then purified using silica gel chromatography gradient eluting with 0–15 % EtOAc/*n*-heptane, giving **30** as a white solid in 54 % yield. **^1^H NMR** (300 MHz, CDCl_3_) δ 7.95–7.88 (m, 2H), 7.63–7.57 (m, 2H), 2.64 (s, 3H). **HPLC** Rt (min) 2.208. **LCMS** (*m/z*) 239.0, 241.0 (M + H^+^).

*3-(4-Bromophenyl)-5-phenyl-1,2,4-oxadiazole (****31****).* Benzoic acid (25 mg, 0.23 mmol), HATU (93 mg, 0.25 mmol) and DIPEA (0.09 mL, 0.51 mmol) were dissolved in DMF (1.5 mL) and stirred at room temperature for 30 min. Subsequently, 4-bromo-*N’*-hydroxybenzimidamide (48 mg, 0.23 mmol, **29**) was added and reaction solution was stirred at 100 ^°^C for 12 h. The solution was cooled to room temperature and concentrated *in vacuo* to give the crude product. The product was then purified using silica gel chromatography gradient eluting with 0–15 % EtOAc/*n*-heptane, giving **31** as a white solid in 49 % yield. **^1^H NMR** (300 MHz, DMSO-*d_6_*) δ 8.23–8.13 (m, 2H), 8.09–7.97 (m, 2H), 7.84–7.78 (m, 2H), 7.77–7.70 (m, 1H), 7.66 (m, 2H). **HPLC** Rt (min) 2.969. **LCMS** (*m/z*) 301.0, 303.0 (M + H^+^).

*5-Methyl-3-(4-(phenylethynyl)phenyl)-1,2,4-oxadiazole (****32****).* 3-(4-Bromophenyl)-5-phenyl-1,2,4-oxadiazole (50 mg, 0.21 mmol, ***31***) was dissolved in DMF (2 mL) and degassed with N_2_. Ethynylbenzene derivative (21 mg, 0.21 mmol), CuI (2 mg, 0.02 mmol) and DIPEA (0.1 mL, 0.6 mmol) were then added to the mixture, which was then degassed with N_2_. Subsequently, Pd(dppf)Cl_2_ (1.5 mg, 0.002 mmol) was added, and the solution was degassed with N_2_ and allowed to stir at 45 ^°^C for 24 h. The reaction was quenched with 1M NH_4_Cl (2.5 mL) and diluted with EtOAc (10 mL). Brine (5 mL) was added, the solution extracted with EtOAc (3 × 4 mL) and the combined organic layers dried over anhydrous Na_2_SO_4_, filtered, and concentrated *in vacuo* to the give the crude product. The product was then purified using silica gel chromatography gradient eluting with 0–40 % DCM/*n*-heptane, giving **32** as a light brown solid in 44 % yield. **^1^H NMR** (300 MHz, DMSO-*d_6_*) δ 8.03 (d, *J* 8.4 Hz, 2H), 7.72 (d, *J* 8.4 Hz, 2H), 7.63–7.54 (m, 2H), 7.49–7.39 (m, 3H), 2.67 (s, 3H).**^13^C NMR** (75 MHz, DMSO-*d_6_*) δ 177.69, 167.10, 132.18, 131.53, 129.20, 128.84, 127.23, 126.23, 125.22, 121.86, 91.54, 88.63, 12.04. **HPLC** Rt (min) 2.741. **LCMS** (*m/z*) 261.2 (M + H^+^). **HRMS acquired**: (M + H^+^) 261.1020; C_17_H_112_N_2_O requires (M + H^+^), 261.1022.

*5-(4-(1H-Pyrazol-5-yl)phenyl)-3-phenyl-1,2,4-oxadiazole (****33****).* 3-(4-Bromophenyl)-5-phenyl-1,2,4-oxadiazole (32 mg, 0.11 mmol, **31**), (1-(*tert*-butoxycarbonyl)-1*H*-pyrazol-5-yl)boronic acid (25 mg, 0.012 mmol) and K_2_CO_3_ (49 mg, 0.36 mmol) were dissolved in a solvent system of 9:1 1,4-dioxane/water (2 mL). The solution was degassed with N_2_ prior to the addition of Pd(dppf)Cl_2_ (4.9 mg, 0.006 mmol), and then allowed to stir at 90 ^°^C for 24 h. The solution was allowed to cool to room temperature and concentrated *in vacuo*. The concentrate was dissolved in EtOAc (5 mL), washed with water (5 mL) and extracted with EtOAc (3 × 5 mL), with the organic layers then being combined, dried over anhydrous Na_2_SO_4_, filtered, and concentrated *in vacuo* to the give the crude product. The product was then purified using silica gel chromatography (5 % MeOH/DCM), giving compound **33** as a white solid in 24 % yield. Note, the *boc* protecting group was inadvertently removed during the reaction. **^1^H NMR** (300 MHz, DMSO-*d_6_*) δ 13.46 (s, 0.2H, NH tautomer), 13.07 (s, 0.8H, NH tautomer), 8.24–8.17 (m, 2H), 8.13 (d, *J* 8.2 Hz, 2H), 8.04 (d, *J* 8.1 Hz, 2H), 7.89–7.54 (m, 4H), 6.85 (d, *J* 2.2 Hz, 1H). **^13^C NMR (**75 MHz, DMSO-*d_6_*) δ 175.43, 168.14, 133.42, 129.63, 127.98, 127.60, 125.81, 124.83, 123.42, 102.58. ^1^H–^13^C HSQC experiment indicates pyrazole **C**-H is obscured (**Additional File 1: Fig. S7**), two quaternary carbons remain unresolved. **HPLC** Rt (min) 2.416. **LCMS** (*m/z*) 289.2 (M + H^+^). **HRMS acquired**: 289.1085 **(**M + H^+^); C_17_H_12_N_4_O requires (M + H^+^), 289.1084.

**Supplementary Figure 6**. The ^1^H,^13^C and ^19^F spectra, and HPLC traces, of UMW-9729 and all analogues.

S1a. ^1^H NMR spectra of compound **1** (DMSO-*d_6_*, 300 MHz).

S1b. ^13^C NMR spectra of compound **1** (DMSO-*d_6_*, 75 MHz).

S1c. Full ^1^H – ^13^C HSQC spectra of compound **1** (DMSO-*d_6_*, 75 MHz).

S1d. Aromatic region of ^1^H – ^13^C HSQC spectra of compound **1** (DMSO-*d_6_*, 75 MHz).

S1e. HPLC trace of compound **1.**

S2a. ^1^H NMR spectra of compound **2** (DMSO-*d_6_*, 300 MHz).


S2b. HPLC trace of compound **2**.

S3a. ^1^H NMR spectra of compound **3** (DMSO-*d_6_*, 300 MHz).


S3b. HPLC trace of compound **3**.

S4a. ^1^H NMR spectra of compound **4** (DMSO-*d_6_*, 300 MHz).


S4b. HPLC trace of compound **4**.

S5a. ^1^H NMR spectra of compound **5** (CDCl_3_, 300 MHz).


S5b. HPLC trace of compound **5**.

S6a. ^1^H NMR spectra of compound **6** (DMSO-*d_6_*, 300 MHz).

S6b. ^13^C NMR spectra of compound **6** (DMSO-*d_6_*, 75 MHz).

S6c. Full ^1^H – ^13^C HSQC spectra of compound **6** (DMSO-*d_6_*, 75 MHz).

S6d. Aromatic region of ^1^H – ^13^C HSQC spectra of compound **6** (DMSO-*d_6_*, 75 MHz).


S6e. HPLC trace of compound **6**.

S7a. ^1^H NMR spectra of compound **7** (DMSO-*d_6_*, 300 MHz).


S7b. ^13^C NMR spectra of compound **7** (DMSO-*d_6_*, 75 MHz).

S7c. ^19^F NMR spectra of compound **7** (DMSO-*d_6_*, 282 MHz).


S7d. HPLC trace of compound **7**.

S8a. ^1^H NMR spectra of compound **8** (DMSO-*d_6_*, 300 MHz).

S8b. ^13^C NMR spectra of compound **8** (DMSO-*d_6_*, 75 MHz).

S8c. ^19^F NMR spectra of compound **8** (DMSO-*d_6_*, 282 MHz).


S8d. HPLC trace of compound **8**.

S9a. ^1^H NMR spectra of compound **9** (DMSO-*d_6_*, 300 MHz).


S9b. ^13^C NMR spectra of compound **9** (DMSO-*d_6_*, 75 MHz).

S9c. Full ^1^H – ^13^C HSQC spectra of compound **9** (DMSO-*d_6_*, 75 MHz).

S9d. Aromatic region of ^1^H – ^13^C HSQC spectra of compound **9** (DMSO-*d_6_*, 75 MHz).


S9e. HPLC trace of compound **9**.

S10a. ^1^H NMR spectra of compound **10** (DMSO-*d_6_*, 300 MHz).

S10b. ^13^C NMR spectra of compound **10** (DMSO-*d_6_*, 75 MHz).

S10c. Full ^1^H – ^13^C HSQC spectra of compound **10** (DMSO-*d_6_*, 75 MHz).

S10d. Aromatic region of ^1^H – ^13^C HSQC spectra of compound **10** (DMSO-*d_6_*, 75 MHz).


S10e. HPLC trace of compound **10**.

S11a. ^1^H NMR spectra of compound **11** (DMSO-*d_6_*, 300 MHz).

S11b. ^13^C NMR spectra of compound **11** (DMSO-*d_6_*, 75 MHz).


S11c. HPLC trace of compound **11**.

S12a. ^1^H NMR spectra of compound **12** (DMSO-*d_6_*, 300 MHz).


S12b. ^13^C NMR spectra of compound **12** (DMSO-*d_6_*, 75 MHz).

S12c. ^19^F NMR spectra of compound **12** (DMSO-*d_6_*, 282 MHz).


S12d. HPLC trace of compound **12**.

S13a. ^1^H NMR spectra of compound **13** (DMSO-*d_6_*, 300 MHz).

S13b. ^13^C NMR spectra of compound **13** (DMSO-*d_6_*, 75 MHz).

S13c. ^19^F NMR spectra of compound **13** (DMSO-*d_6_*, 282 MHz).


S13d. HPLC trace of compound **13**.

S14a. ^1^H NMR spectra of compound **14** (DMSO-*d_6_*, 300 MHz).


S14b. ^13^C NMR spectra of compound **14** (DMSO-*d_6_*, 75 MHz).

S14c. Full ^1^H – ^13^C HSQC spectra of compound **14** (DMSO-*d_6_*, 75 MHz).

S14d. Aromatic region of ^1^H – ^13^C HSQC spectra of compound **14** (DMSO-*d_6_*, 75 MHz).

S14e. ^19^F NMR spectra of compound **14** (DMSO-*d_6_*, 282 MHz).

S14f. HPLC trace of compound **14**.

S15a. ^1^H NMR spectra of compound **5** (DMSO-*d_6_*, 300 MHz).

S15b. ^13^C NMR spectra of compound **15** (DMSO-*d_6_*, 75 MHz).


S15c. HPLC trace of compound **15**.

S16a. ^1^H NMR spectra of compound **16** (DMSO-*d_6_*, 300 MHz).


S16b. ^13^C NMR spectra of compound **16** (DMSO-*d_6_*, 75 MHz).

S16c. HPLC trace of compound **16**.

S17a. ^1^H NMR spectra of compound **17** (DMSO-*d_6_*, 300 MHz).


S17b. ^13^C NMR spectra of compound **17** (DMSO-*d_6_*, 75 MHz).

S17c. HPLC trace of compound **17**.

S18a. ^1^H NMR spectra of compound **18** (DMSO-*d_6_*, 300 MHz).

S18b. ^13^C NMR spectra of compound **18** (DMSO-*d_6_*, 75 MHz).


S18c. HPLC trace of compound **18**.

S19a. ^1^H NMR spectra of compound **19** (DMSO-*d_6_*, 300 MHz).


S19b. ^13^C NMR spectra of compound **19** (DMSO-*d_6_*, 75 MHz).


S19c. HPLC trace of compound **19**.

S20a. ^1^H NMR spectra of compound **20** (DMSO-*d_6_*, 300 MHz).

S20b. ^13^C NMR spectra of compound **20** (DMSO-*d_6_*, 75 MHz).

S20c. HPLC trace of compound **20**.

S21a. ^1^H NMR spectra of compound **21** (DMSO-*d_6_*, 300 MHz).

S21b. ^13^C NMR spectra of compound **21** (DMSO-*d_6_*, 75 MHz).

S21c. Full ^1^H – ^13^C HSQC spectra of compound **21** (DMSO-*d_6_*, 75 MHz).

S21d. Aromatic region of ^1^H – ^13^C HSQC spectra of compound **21** (DMSO-*d_6_*, 75 MHz).


S21e. HPLC trace of compound **21**.

S22a. ^1^H NMR spectra of compound **22** (DMSO-*d_6_*, 300 MHz).


S22b. ^13^C NMR spectra of compound **22** (DMSO-*d_6_*, 75 MHz).


S22c. HPLC trace of compound **22**.

S23a. ^1^H NMR spectra of compound **23** (DMSO-*d_6_*, 300 MHz).


S23c. HPLC trace of compound **23**.

S24a. ^1^H NMR spectra of compound **24** (DMSO-*d_6_*, 300 MHz).

S24b. ^13^C NMR spectra of compound **24** (DMSO-*d_6_*, 75 MHz).

S24c. Full ^1^H – ^13^C HSQC spectra of compound **24** (DMSO-*d_6_*, 75 MHz).

S24d. Aromatic region of ^1^H – ^13^C HSQC spectra of compound **24** (DMSO-*d_6_*, 75 MHz).


S24e. HPLC trace of compound **24**.

S25a. ^1^H NMR spectra of compound **25** (DMSO-*d_6_*, 300 MHz).


S25b. ^13^C NMR spectra of compound **25** (DMSO-*d_6_*, 75 MHz).


S25c. HPLC trace of compound **25**.

S26a. ^1^H NMR spectra of compound **26** (DMSO-*d_6_*, 300 MHz).


S26b. HPLC trace of compound **26**.

S27a. ^1^H NMR spectra of compound **27** (DMSO-*d_6_*, 300 MHz).

******

S27b. ^13^C NMR spectra of compound **27** (DMSO-*d_6_*, 75 MHz).

S27c. Full ^1^H – ^13^C HSQC spectra of compound **27** (DMSO-*d_6_*, 75 MHz).

S27d. Aromatic region of ^1^H – ^13^C HSQC spectra of compound **27** (DMSO-*d_6_*, 75 MHz).


S27e. HPLC trace of compound **27**.

S28a. ^1^H NMR spectra of compound **28** (CDCl_3_, 300 MHz).

S28b. HPLC trace of compound **28**.

S29a. ^1^H NMR spectra of compound **29** (DMSO-*d_6_*, 300 MHz).

S29b. HPLC trace of compound **29**.

S30a. ^1^H NMR spectra of compound **30** (DMSO-*d_6_*, 300 MHz).

S30b. HPLC trace of compound **30**.

S31a. ^1^H NMR spectra of compound **31** (DMSO-*d_6_*, 300 MHz).

S31b. HPLC trace of compound **31**.

S32a. ^1^H NMR spectra of compound **32** (DMSO-*d_6_*, 300 MHz).

S32b. ^13^C NMR spectra of compound **32** (DMSO-*d_6_*, 75 MHz).

S32c. HPLC trace of compound **32**.

S33a. ^1^H NMR spectra of compound **33** (DMSO-*d_6_*, 300 MHz).

S33b. ^13^C NMR spectra of compound **33** (DMSO-*d_6_*, 75 MHz).

S33c. Full ^1^H – ^13^C HSQC spectra of compound **33** (DMSO-*d_6_*, 75 MHz).

S33d. Aromatic region of ^1^H – ^13^C HSQC spectra of compound **33** (DMSO-*d_6_*, 75 MHz).

S33e. HPLC trace of compound **33**.
